# Species delimitation of neotropical Characins (Stevardiinae): Implications for taxonomy of complex groups

**DOI:** 10.1371/journal.pone.0216786

**Published:** 2019-06-05

**Authors:** Jorge E. García-Melo, Claudio Oliveira, Guilherme José Da Costa Silva, Luz E. Ochoa-Orrego, Luiz Henrique Garcia Pereira, Javier A. Maldonado-Ocampo

**Affiliations:** 1 Laboratorio de Ictiología, Unidad de Ecología y Sistemática (UNESIS), Departamento de Biología, Facultad de Ciencias, Pontificia Universidad Javeriana, Bogotá, Colombia; 2 Departamento de Morfologia, Instituto de Biociências, Universidade Estadual Paulista (UNESP), Campus de Botucatu, Botucatu, SP, Brazil; 3 Universidade Santo Amaro, Rua Prof. Enéas de Siqueira Neto, Jardim das Imbuias, São Paulo—SP, Brazil; 4 Centro de Ciências da Vida e da Natureza, Universidade Federal da Integração Latino-Americana–UNILA, Foz do Iguaçu, Paraná, Brazil; SOUTHWEST UNIVERSITY, CHINA

## Abstract

Accurate species delimitation is crucial for studies of phylogeny, phylogeography, ecology, conservation and biogeography. The limits of species and genera in the Characidae family are controversial due to its uncertain phylogenetic relationships, high level of morphological homoplasy and the use of ambiguous morphological characters for descriptions. Here we establish species boundaries for *Bryconamericus*, *Hemibrycon*, *Knodus* and *Eretmobrycon* (Stevardiinae: Characidae), previously diagnosed with morphology, using three different barcoding approaches (GMYC, PTP, ABGD). Results revealed that species delimitation was successful by the use of a single-gene approach and by following a workflow in the context of integrative taxonomy, making evident problems and mistakes in the cataloging of Characidae species. Hence, it was possible to infer boundaries at genus level for clusters in the trees (GMYC and PTP) and automatic partitions (ABGD) which were consistent with some of recent taxonomic changes proposed in Characidae. We found that discordance cases between methods were linked to limitations of the methods and associated to putative species cluster closely related, some historically problematic in their diagnosis and identification. Furthermore, we suggested taxonomic changes and possibly new species, revealing a high degree of hidden diversity. Finally, we propose a workflow as a fast, accurate and objective way to delimit species from mitochondrial DNA sequences and to help clarify the classification of this group.

## Introduction

The accelerated loss of biodiversity and the high number of species that have yet to be described have generated a ‘taxonomy crisis’ and the need for more effective ways to discover and delimitate species [1]. Classification and phylogeny rely on species as their primary unit of research, and therefore, inaccurate delimitations or unidentified species can seriously affect biodiversity assessments, the understanding of evolutionary relationships and the validity of comparative studies [2]. Accurate species delimitation is a challenge specially for cryptic taxa [3,4] where characters used to define species are uninformative or incongruent [5], in such cases, traditional morphological studies are an intensive labor and difficult to apply for group or species complexes that are externally indistinguishable based on morphological traits [6–8].

Currently, a general consensus among taxonomists is that an ‘integrative’ or ‘iterative’[9] taxonomic approach including multiple lines of evidence (e.g. behavioral, ecological, molecular, morphological) is the best way for accurate species delimitation. Thus, methods based on DNA are creating a tremendous opportunity for taxonomists and biodiversity scientists to delimit putative species (i.e., operational taxonomic units, OTUs) [1,10]. However, despite the undisputed theoretical superiority of multi-locus studies for inferring species boundaries [3,4,11], single-locus data provide a practical and fast approach for studies of hundreds or even thousands of species simultaneously [12–15], at low cost and counting with a Primary Species Hypotheses (PSH) or candidate species.

Recent DNA-based studies suggest that tropical faunas contain a large proportion of undescribed species, which can result in underestimated species-level diversity [16], especially freshwater fishes of the order Characiformes that show high levels of cryptic diversity [7,17,18]. Within Characiformes, Characidae is one of the largest and heterogeneous families of Neotropical fishes, with approximately 1172 valid species in approximately 146 genera [19]. The discovery and description of Characins species have been challenging, with more than 236 species for the last 10 years [19], but this has been primarily based on traditional morphology and morphometric analysis that use highly homoplastic characteristics that are insufficient to diagnose generic or suprageneric clades [20,21]. Moreover, morphologically established limits of species and genera within this family are controversial due to uncertain phylogenetic relationships [22,23] and the use of ambiguous morphological characters for descriptions not based in autapomorphies.

Genera such as *Bryconamericus* (55 species), *Hemibrycon* (51 species), *Knodus* (36 species) and *Eretmobrycon* (12 species) occur from lower Central America to western Argentina [24], and currently, they are placed in the subfamily Stevardiinae [25], even though historically their actual placement has been controversial due to the lack of morphological diagnosable characteristics, morphological similarities between species and high intraspecific variation [26]. This ambiguous diagnosis has generated confusion in species descriptions and taxonomic keys to species identifications. This is evident in the high number of taxonomic uncertainties (species cataloged as ‘sp.’, ‘aff.’ and ‘cf.’) and misidentified or uncataloged species in ichthyology collections. Thus, multiple records are assigned by geographic occurrence rather than a deep revision of morphological descriptions, and this problem is magnified when there are cryptic, sympatric [27], or complex species in the same hydrogeographic basin [26]. Therefore, the current number of valid species and their distribution for these genera remains uncertain [28–31].

Considering the delimitation species issues in *Bryconamericus*, *Eretmobrycon*, *Hemibrycon* and *Knodus* based on morphologic characteristics, the aims of the present study were to compare the putative species recovered using species delimitation models based on genetic data (GMYC, PTP, ABGD) with those established by morphology alone and to discuss how methods with different assumptions can help with taxonomy and species delimitation within the Stevardiinae group. Additionally, we proposed an objective workflow (pipeline) in the context of integrative taxonomy to classify Neotropical fishes in ichthyological collections.

## Methods

### Study material and focal taxa

In this study we evaluated 382 specimens of Characidae deposited in the fish collections of six institutions (S1 Table). We had access to 312 specimen tissues that were sequenced for barcoding (Cytochrome c Oxidase I or COI), 250 of which corresponded to morphospecies of our focal taxa: the genera *Bryconamericus*, *Eretmobrycon* (*Lato sensu* [31]), *Knodus* and *Hemibrycon*. Sequences of type species of *Eretmobrycon* (*E*. *bayano*), *Bryconamericus* (*B*. *exodon*) and *Knodus* (*K*. *meridae*) were used in the analysis to delineate the genera in the resulting clusters. The sampling strategy included a large number of specimens (full dataset), covering a wide range of basins in order to increase the probability of sampling closely related species and to not overestimate the interspecific differences [32]. These species are distributed in the Lower Central America and trans and cis-Andean regions (i.e. Bayano, San Juan, Atrato, Magdalena-Cauca, Orinoco, Amazonas, La Plata, basins among others). Barcoding sequences of closely related genera also were included and DNA data set generated in this study was deposited into Genbank (accession numbers: MH002916—MH003295).

### DNA extraction and sequencing

Total genomic DNA was isolated from muscle tissues with a DNeasy Tissue Kit (Qiagen), according to the manufacturer’s instructions. Amplifications were performed in a total volume of 12.5 μl, with 1.25 μl of 10X buffer (10mM Tris-HCl+15mM MgCl2), 0.5 μl dNTPs (200 nM of each), 0.5 μl each 5mM primer (L6252-Asn, H7271-COXI described in [33]), 0.25 U Platinum *Taq* Polymerase (Invitrogen), 1 μl template DNA (12 ng), and 8.7 μl ddH2O. PCR reactions consisted of 30–40 cycles for 30 s at 95°C, 15–30 s at 48–54°C, and 45 s at 72°C. All PCR products were first visually identified on a 1% agarose gel and then purified using ExoSap-IT (USB Corporation), following the manufacturer’s instructions and the purified PCR products were sequenced using a Big Dye Terminator v 3.1 Cycle Sequencing Ready Reaction Kit (Applied Biosystems), purified again by ethanol precipitation and loaded onto an automatic sequencer 3130-Genetic Analyzer (Applied Biosystems). Afterwards, consensus sequences from forward and reverse strands were obtained using Geneious Pro 4.8.5 [34] and BioEdit [35] software. Alignments were generated using Muscle [36] under default parameters and the alignment was inspected by eye for any obvious misalignments such as sequencing errors due to contamination, paralogy or pseudogenes. Nucleotide variation, substitution patterns and genetic distances were examined using the BOLD system tools. To evaluate the occurrence of substitution saturation, we estimated the Iss (Index of substitution saturation) in DAMBE 5.2.3 [37], as described by Xia et al.[38], and the rate of transitions/transversions was also evaluated with this software.

### Phylogenetics analysis

The best nucleotide evolution models for the COI gene were evaluated using PartitionFinder v1.1.0 [39] under the information-theoretical measure of Akaike Information Criterion (AICc). To generate the best maximum likelihood (ML) tree, the RAxML PTHREADS‐SSE3 implemented in RAxML v8.019 [40] was performed on 2 Å~ 10 CPU, 256 GB Zungaro server at IBB‐UNESP. RAxML executed five inferences on the original data set using five distinct randomized maximum‐parsimony topologies through the GTRGAMMA model [41], and 1,000 nonparametric bootstrap replicates using the MRE‐based bootstopping criterion, resulting in a total of 600 pseudoreplicates.

### Species delimitation and recognition of putative species

**Fig 1:**
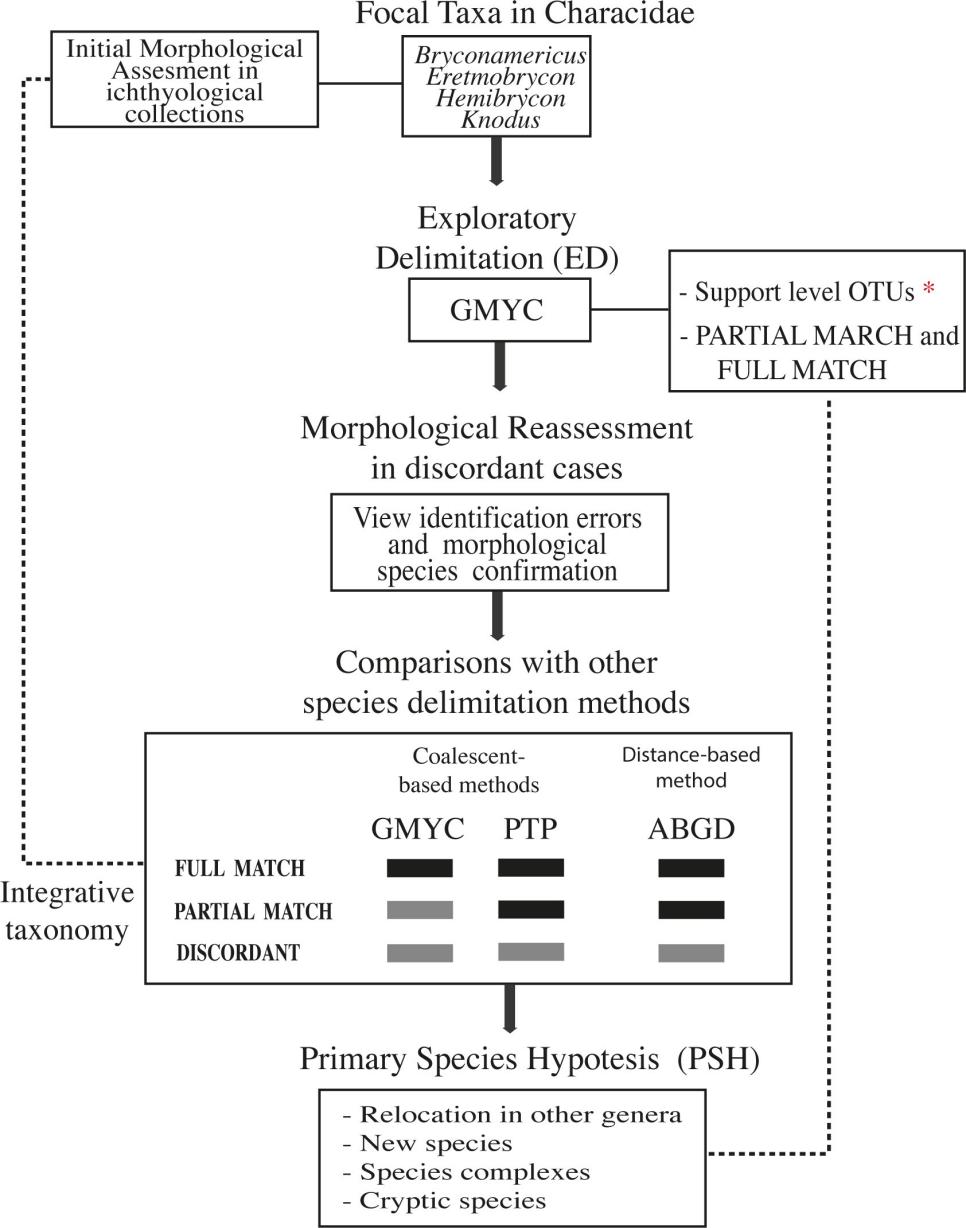
Objective workflow (pipeline) used in the context of integrative taxonomy to delimitate Characins in ichthyological collections. Black and grey squares represent the possible rearrangements for integrative taxonomy. Full match, by which the three molecular methods generated the same grouping; Partial Match by which at least two of the molecular methods generated the same grouping; and Discordant, by which all three molecular methods led to a different result.

To assign individuals to species and to develop an initial delimitation, we sorted species based in collection morphology (Initial Morphological Assessment, Fig 1). The generalized mixed Yule-coalescent GMYC method [42] allows to determine the point of transition from species-level to population-level combining models of stochastic lineage growth with coalescent theory [43] in a robust maximum-likelihood generating a ultrametric guide tree. We used this method as the main approach to exploratory delimitation (ED) of species boundaries based on DNA barcodes and to establish OTUs due to it is frequently used in empirical studies has been and outperform OTU-picking methods (relying on simple sequence similarity thresholds) and are more robust to cases where the barcoding gap is absent [44,45]. This method is useful when multiple individuals belong to putative species are analyzed [46]. In spite previous studies have found that in some cases GMYC could lead to an overestimation of the number of species [47], paradoxically, this background makes it an appropriate method to evaluate the largest number of a priori groups in the exploratory analysis and to compare with regularly more conservative methods [10,47]. This model has been focus of multiples controversy, in relation to the dataset, in this case content a dataset with many species of the which several are represented only by singletons or few specimens, it was already proved that this model is more appropriated than Gaussian clustering in this kind of dataset [48]. After the GMYC analysis, OTUs were reassessed morphologically in specimens for which we had access to the vouchers in order to shows obvious identification errors and morphological species confirmation. Furthermore, we compared with other species delimitation methods: Poisson Tree Processes (PTP) [44] and Automatic Barcode Gap Discovery (ABGD; [49]) (Fig 1). Thus, the percentage of congruence between the methods was obtained considering the total number of concordances with respect to the total number of comparisons.

To conduct the GMYC model analysis, we estimated an ultrametric tree in BEAST v.1.6.2 [50] using a lognormal relaxed molecular clock and GTR model with Gamma distribution and birth-death speciation process rate on an arbitrary timescale, and a random UPGMA tree was used as the starting tree for the Markov chain Monte Carlo searches. The length of MCMC chain was 20 000 000 sampling every 10000. The convergence of analysis was evaluated in Tracer v1.6.0 [51] according of the ESS values and the maximum clade credibility tree was built from the combined runs after eliminating 20% of the trees for burn-in in TreeAnnotator v1.7.2. The GMYC approach was carried out in R 3.0.0 [52] using the splits (Species Limits by Threshold Statistics [53] and ape (Analyses of Phylogenetics and Evolution in R language [54] packages using the “single threshold” model [55]). In the Poisson tree processes (PTP) the Yule-coalescent transition points are modeled based on the change of substitution rates on the phylogenetic input tree. We used the best ML tree as input data and the calculations were subjected to bPTP websever (http://species.h-its.org/ptp/), with 500,000 MCMC generations, thinning set to 100 and burnin at 10% and performing a maximum likelihood solution. We conducted two runs: the first one, without removing the sequences that were allocated outside of Stevardiinae in the GMYC analysis; and second, considering them as “Outgroup” and therefore removing them to improve the delimitation results of focal taxa.

The ABGD analysis was performed on the ABGD website (http://wwwabi.snv.jussieu.fr/public/abgd/abgdweb.html) with P (prior intraspecific divergence) set from 0.001 to 0.1 (refer to the area were the barcode gap should be detected) and Steps set to 10; X (minimum relative gap width, i.e. relates to the sensitivity of the method to gap width) set to 1, Nb bins (for distance distribution) set to 20 and we selected the Kimura (K80) model [56]. The gap could be considered as the threshold of the upper limit of intraspecific distances and the lower limit of interspecific distances.

Posterior probabilities (PP; ≥ 0.95) for trees obtained by GMYC and PTP methods were considered to assess the level of confidence limits of each node in the recovered OTUs. The three methods were compared to test the congruence between them and to evaluate possible taxonomic overestimation (taxonomic over-splitting) [57] or taxonomic underestimation.

### Initial morphological assessment in ichthyological collections

Specimens deposited in the fish collections had been subject to four types of cataloging: assigned species, “sp.” (species indeterminata), “aff.”(species affinis) and “cf.” (confer; compare). The initial morphological assessment had been performed by ichthyologists who cataloged the species in each of the biological collections (S1 Table), however, almost 50% of the total analyzed data set were taxonomic uncertainties, which is relatively common to several Characidae genera. To ensure accurate species identification, the trans-Andean specimens of *Bryconamericus* and *Hemibrycon* deposited at MPUJ were collected at their type localities. In addition, the cartilage and skeletons were subject to staining [58] and the specimens were determined to species level using the available original descriptions and taxonomic keys [59–86].

### Integrative taxonomy

If we assume that species represent ‘segments of metapopulation lineages’ [87], then direct genetic evidence of lineage status is particularly relevant to species delimitation studies when analyzed within a rigorous statistical framework, regardless of whether lineages differ in phenotypic characters that are apparent to human observers [88]. Our initial approach was to look for different species, considering all available specimens identified as described above; these specimens correspond to different morphospecies (species identified under a morphological framework considering all available literature). Based on this initial step, we used an iterative process to compare all sequenced specimens and determine species boundaries under different approaches. A general workflow modified from [10] and [89] for the species delimitation decision-making process is provided in Fig 1. Here, three sequence-based methods of species delimitation were used to delimitate species, and a final delimitation criteria was established based on a 66% consensus among methods (congruence in at least 2 methods or Partial Match) and the support for groups (GMYC and PTP), by which posterior probabilities greater than or equal to 0.95 accept the grouping, and those less than 0.95 reject it. Finally, BIN (Barcode Index Number) analysis was carried out automatically in the BOLD System on the specimens for which it was not possible to conduct a morphological re-examination, with doubtful positions in the tree. This facilitated detection of possible errors in the tissue cataloging process. The same process was also performed for samples of the same species originating from the same locality or same lot, which also exhibited inconsistencies. By assigning specimens to OTUs that closely approximate species and by aggregating collateral data, the BIN system illuminates species diversity from groups which have seen little taxonomic investigation [14].

## Results

We analyzed barcode sequences for 382 specimens with more than 500 base pairs. After alignment and editing, the final matrix had 521 characters, of which 211 positions were conserved and 310 were variable. Base composition for this fragment was 24.4% adenine, 26.3% cytosine, 31.3% thymine and 17.5% guanine. Considering that the Iss.c value was greater than the Iss, the data were not saturated, and the R2 value was greater than 0.74 for transitions and transversions (S1 Fig). The optimal gene trees produced by neighbor−joining (NJ) Kimura 2 Parameter and ML analysis showed nearly similar species delimitation in which the branch tips within OTUs were short, and OTUs were separated by longer branches (S2 Fig and S3 Fig).

### Exploratory delimitation (ED)

**Fig 2:**
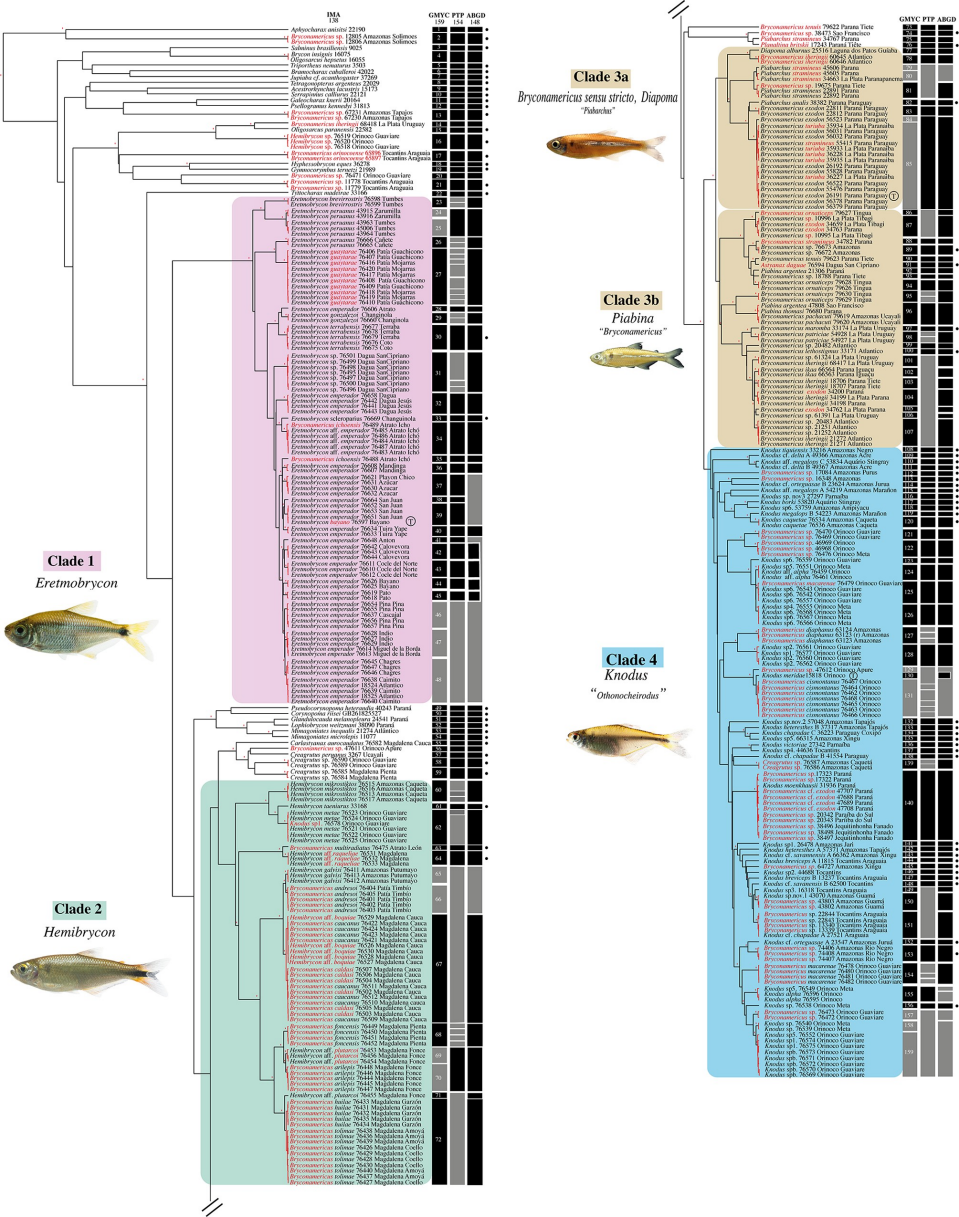
Bayesian phylogenetic tree of *Eretmobrycon*, *Bryconamericus*, *Hemibrycon* and *Knodus* obtained with COI data. The red asterisks in the node branches represent a posterior probability higher than 95%, and the red terminal branches in the tree show the transition point from Yule to a coalescent branching process in the analysis of all sequences as estimated by the single-threshold model in the GMYC test. The first column represents monophyletic clusters recovered by GMYC analysis, and the second and third columns correspond to the status of the delimitation of OTUs by PTP and ABGD. Full match, Partial Match and Discordant categories between the three molecular methods are represented by black and grey squares. OTUs recovered with all methodologies including initial morphological assessment are indicated with a black circle. Identification errors and suggested changes or taxonomic confirmations (to genus and/or species levels) are highlighted in red letters. The red asterisk in the nodes is indicating PP ≥0.95. The letter T indicates the type species of the genus.

The results of ED with GMYC delimitations were compared to the initial morphological assessment. The ED recovered at least 159 OTUs or putative species clusters (first column, Fig 2) from 382 specimens sampled from 140 morphospecies of Characidae, with a confidence interval of 138 to 171 for the tree based on a relaxed lognormal clock. The threshold obtained (time at which the model infers that the threshold transitioning from the speciation-level events to the coalescent-level events takes place) was -4.51x10^-3T^, where T = time from present to the time of the root (S4 Fig). The likelihood of the GMYC model in the analysis of the mitochondrial gene COI was 3200.263, and it was significantly superior to the likelihood of the null model (L0 = 3130.468, P−value = 0.01). The ED confirmed the identity of 91 of 142 morphospecies; this represents, the correspondence between the initial morphological assessment and putative species clusters recovered by GMYC analysis, which are consistent with the limits of the studied genera. The GMYC tree showed high support for the terminal nodes (Posterior Probability, PP; ≥0.95) and low support for the basal nodes (S1 File). As expected, not all morphospecies were monophyletic. To facilitate visualization of the results, we established distinctly monophyletic clades (highlighted in Fig 2, Clade 1, Clade 2, Clade 3a, Clade 3b, Clade 4) based on the results of ED.

On the other hand, some morphospecies are not congruent with the ED (highlighted in red letters, Fig 2) suggesting the existence of problems and errors in the identification of species of the genera studied.

### Identification errors

Exploratory delimitation (ED) and morphological reassessment of 244 vouchers reassigned 85 specimens to other genera in seven ichthyological collections (Table 1). The main misidentifications were found in species grouped into clades 3a, 3b and 4 (Fig 2). We found sixty erroneous identifications in individuals previously allocated in the genus *Bryconamericus*. These individuals did not correspond to subfamily Stevardiinae (OTUs 2, 13, 14, 16, 17, 20 and 21 from the top of the tree, Fig 2) and were allocated in corresponded to distantly related species such as the genera *Prionobrama*, *Bryconops* and *Hemigrammus*.

**Table 1:** Number of specimens reassigned to other genera after ED and morphological reassessment.

Cataloged in ichthyological collections as:	Genera reassigned after ED and morphological reassessment	Number of specimens
*Bryconamericus*	*Eretmobrycon*	2
	*Bryconamericus*	22
	*Knodus*	43
	*Diapoma*	2
	*Piabina*	2
	*Lepidocharax*	2
	*Creagrutus*	1
	*Bryconops*	1
	*Planaltina*	2
	*Hemmigrammus*	1
	Out of Stevardiinae	3
*Creagrutus*	*Othonocheirodus*	2
*Hemibrycon*	Out of Stevardiinae	2

Tissues with possible cataloging errors were detected (S1 Table) when the geographic distribution was revised. These corresponded to *Astyanax daguae* from Dagua river, (GMYC OTU 91) and *Bryconamericus pachacuti* from Amazonas Ucayali basin (GMYC OTU 96). Also, specimens of the same lot are allocated distantly within the tree, showing that lots have been mixed with different species or even genera. Examples are *Bryconamericus* sp. Guaviare (GMYC OTU 20) and *Bryconamericus* sp. Guaviare (GMYC OTU 121). An analogous situation occurs within the same genus for specimens of *Knodus alpha* (GMYC OTU 155,124) and *Knodus macarenae* (GMYC OTU 125,124) in Clade 4.

*Bryconamericus exodon* (the type species of *Bryconamericus*), *B*. *iheringii* and *Piabarchus stramineus* were the species with the most taxonomic corrections after the morphological reassessment. Most *Knodus* species identification errors correspond to species cataloged as *Bryconamericus* (Fig 2, S1 Table). In this clade, a species identified as *Creagrutus* sp. from the Caquetá-Amazon Basin corresponds to *Othonocheirodus* sp.

Exploring our data set throughout BIN analysis also showed that there are lots with mixtures of specimens of different species; such as the case of *Bryconamericus ornaticeps* (GMYC OTUs 86,94,95), which contains specimens of *Piabina argentea*. In the same way, submitted sequences of *Eretmobrycon guaytarae* matched to *E*. *dahli*, a congeneric species that has not yet been analyzed.

### Congruence between methods

For our dataset, GMYC and PTP yield similar number of splits, while ABGD yields a more conservative delimitation closed to the number of morphological species (Fig 2). We only considered results from the initial partition with K2P for ABGD and maximum likelihood results for PTP, due to the OTUs count were similar when comparing all the methods. Thus, the number of putative species delimited for focal taxa reached 65.4% among the three molecular delimitation methods.

In the first run of PTP procedure, only 29 putative species were recovered, of which 22 correspond to those sequences located outside of Stevardiinae (“Outgroup”) in the GMYC analysis, constituting a total congruence only for these groupings with the other molecular methods. The seven remaining putative species grouped sequences of related species (tribes or genera), but they clearly correspond to erroneous delimitations due to the inclusion of distantly related species sequences (S6 Fig and S4 File).

In the PTP analysis we removed the outgroup and this method recovered 132 putative species, which added to 22 correspond to 154 entities based on the best-fit ML tree (S7 Fig and S4 File). Compared with exploratory analysis, PTP generates more splits in over-sampled morphospecies (second column, Fig 2); for instance, GMYC OTUs 26/27 and 31 of *Eretmobrycon* clade, GMYC OTUs 60, 62 and 68 of *Hemibrycon* clade and GMYC OTUs 131 and 154 of *Knodus* clade, but with low support values (0.5) (S4 File). On the other hand, PTP generates less splits that GMYC analysis in some morphospecies or OTUs closely related. Matches between the two methods were 109 OTUs (i.e. more than 70%), indicating good congruence between the two coalescent-based methods.

ABGD recovered 148 OTUs, of which 136 matched with GMYC OTUs (S8 Fig and Fig 2). Some OTUs identify by GMYC and PTP were clustered in a single OTUs, such as *Eretmobrycon peruanus* (from Zarumilla and Tumbes basin). A similar situation occurs in the clade 2 between *Hemibrycon* aff. *plutarcoi* and *H*. *arilepis* (GMYC OTUs 69 and 70) and between *H*. *galvisi* and *H*. *andresoi* (GMYC OTUs 65 and 66) (Fig 2). In clade 4, ABGD also recovered GMYC OTUs 129 and 131 as one single entity. Compared with the other analysis, only one situation is presented where ABGD divided into more than one OTUs (GMYC OTU 155). Finally, we observed incongruences for the three methods in few cases, mainly associated with morphospecies that geographically are very related within the same or in adjacent basins.

### Integrative taxonomy of focal taxa and PSH

The results from GMYC and PTP trees with inclusion of the type species (T) allowed delimit the genera *Eretmobrycon* (Clade 1), *Hemibrycon* (Clade 2), *Bryconamericus* (Clade 3a, 3b) and *Knodus* (Clade 4). *Bryconamericus* is one of most difficult to define due to this grouping with species of *Piabarchus*, *Diapoma* and *Piabina*. These results are consistent with some of the taxonomic changes proposed by Thomaz et al. [31] and suggest that several species should be reassigned (Fig 2, Table 2).

**Table 2:** New classification suggested and confirmed by delimitation species.

Taxonomic current status according to Eschmeyer et al. 2018 [25]	Taxonomic status suggested	Observation
*Bryconamericus andresoi* Román-Valencia 2003	*Hemibrycon andresoi*	Taxonomic change proposed by [104] is confirmed.
*Bryconamericus arilepis* Román-Valencia, Vanegas-Ríos and Ruiz-C. 2008	*Hemibrycon arilepis*	Taxonomic change proposed by [104] is confirmed.
*Bryconamericus cismontanus* Eigenmann 1914	*Knodus cismontanus*	* *
*Bryconamericus diaphanus* (Cope 1878)	*Knodus diaphanus*	*Incertae sedis* according to [31]
*Bryconamericus foncensis* Román-Valencia, Vanegas-Ríos and Ruiz-C. 2009	*Hemibrycon foncensis*	Taxonomic change proposed by [104] is confirmed.
*Bryconamericus ichoensis* Román-Valencia 2000	*Eretmobrycon ichoensis*	* *
*Bryconamericus macarenae* Román-Valencia, García-Alzate, Ruiz-C. and Taphorn 2010	*Knodus macarenae*	* *
*Bryconamericus multiradiatus* Dahl 1960	*Hemibrycon multiradiatus*	* *
*Bryconamericus tolimae* (Eigenmann 1913)	*Hemibrycon tolimae*	Taxonomic change proposed by [104] is confirmed.
		
	**Synonymous species proposed**	
*Eretmobrycon dahli* Román-Valencia 2000	*Eretmobrycon guaytarae* Eigenmann and Henn, 1914	Not examined by [31]
*Bryconamericus caldasi* Román-Valencia, Ruiz-C., Taphorn B. & García-Alzate 2014	*Hemibrycon cuacanus*	*Incertae sedis* according to [31]
*Bryconamericus huilae* Román-Valencia 2003	*Hemibrycon tolimae*	
*Bryconamericus turiuba* Langeani, F., Z. M. S. de Lucena, J. L. Pedrini and F. J. Tarelho-Pereira 2005	*Bryconamericus exodon*	

According to integrative taxonomy analysis we found 72 OTUs recovered with all methodologies including initial morphological assessment (Fig 2), so the 45.2% of the delimited species were in accordance with genetic and morphological criteria, which was close to the unified species concept proposed by de Queiroz [90] and resulted in clearly delimited putative species in all four clades. The OTUs recovered by molecular methods (all the boxes of the columns in black), evidenced ambiguities in the morphological sorting.

Considering the level of support for each OTU and a 66% consensus among methods, we rejected some putative species and reconsider its assignment (S1 Table). For example, the GMYC analysis recovered three putative species for *E*. *peruanus*, but based on the congruence of the methods of PTP and ABGD (OTU GMYC 24 and 25), we accepted the hypothesis of two putative species distributed in rivers draining into the Pacific in northern Ecuador. Delimitation methods largely converged in identifying numerous OTUs within the morphospecies *Eretmobrycon emperador* from lower Central America (36 to 48) questioning the validity of a single putative species with wide distribution. Despite these groupings, other OTUs that include specimens of *E*. *emperador* from the Atrato (GMYC OTU 34) and Dagua rivers (GMYC OTU 32) are congruent between methods and probably constitute different species. In Clade 3a, a group of morphologically reassessment specimens were considered as *Bryconamericus iheringii*, previously assigned to species, as *B*. *ikaa* and *B*. *patriciae*. GMYC and ABGD analysis retrieves different OTUs for these specimens and we establish the ‘Iheringii complex’, which is composed of species of *Bryconamericus* distributed in the Paraná Basin and Atlantic Ocean drainages. In Clade 4, three GMYC OTUs (157, 158 and 159) composed of *Knodus* morphospecies from the Orinoco river Basin are not supported by the groups established in the ABGD and PTP analysis, and therefore we consider that they can constitute a complex of species.

## Discussion

Our study reveals that the species delimitation obtained for some of the most speciose genera in Characidae support previous hypotheses related to the underestimation of the Neotropical fauna [7,16,18,91]. Our proposal represents one of the first approaches to delimit species in a large set of samples with taxonomic uncertainties–this is “sp.” (species indeterminata), “aff.” (species affinis) and “cf.” (confer; compare)–in ichthyological collections. These taxonomic ambiguities or identification errors are numerous, so implementing methods that can help to solve these errors provide an unique framework to address subsequent questions in taxonomy, systematics and biogeography. It is important to note that the results are the initial evidence of the taxonomic issues discussed earlier in this study such as 1) genera and species of questionable validity; 2) synonymy with other species; 3) cryptic/complexes species and 4) species that should be relocated in other genera (Table 1, Fig 2).

However, the approach and dataset implement here are not exempt of limitations. First, the absence of voucher for some samples analyzed, difficult to perform a morphological reassessment. We found that some ichthyological collections contain in their cryovials whole specimens in alcohol, due to the reduced size of some species. Regularly, this precludes a morphological revision, unless they have specimens of the same morphotype and lot in formaldehyde. Second, the congruence between the molecular methods was not always the best, particularly in complexes of species. This may suggest differing degrees of statistical power inherent to implicit assumptions of every method [92]. For instanced or population sizes and speciation rates can affect the delimitation [93]. As well, for single locus methods base in distance and coalescent- methods (e.g. mtDNA) have been widely documented limitations [94]. Meanwhile, coalescent-based methods depend on the input phylogenetic trees and the priors set in the MCMC analysis [44,95].

According to Kekkonen et al. [96], cases of discordance between the boundaries of reference species and OTUs also can reflect errors by insufficient knowledge of the focal taxa. For instance, in small characins, recurring errors during the identification and sorting process in biological collections can be due to the degree of expertise of the ichthyologist on the taxonomy, anatomy and characteristics recognition of these groups of fish. Additionally, in several cases, the identity of the specimens is defined by the collection locality without an examination of the specimens [97].

### Performance of workflow

Our workflow focused on species delimitation of a large-scale dataset with numerous taxonomic problems. Therefore, the initial morphological assessment used a priori taxonomic assignment of individuals to species and genera deposited in biological collections. In this way, the choice of a method for exploratory delimitation (ED) depended on ability to evaluate the entire data set and visualizing genetic clusters congruent with the initial recognition of genera within Stevardiinae. In this way, it is possible the morphological reassessment considering a priori grouping that include both tree topology and taxonomic assignment uncertainties.

Although some studies in other animal groups have found that GMYC method result in lineages over-splitting relative to the other methods [98,99], this approach enabled the generation of a consistent guide tree. Even when GMYC requires a fully-resolved ultrametric chronogram as input which make it compute-intensive and potentially error-prone process [94], we conclude that was the most practical method for ED of our focal taxa. For large trees (>100 taxa) with samples that potentially correspond to distant species ("outgroup"), the GMYC method was congruent with morphological assessment a priori, comparatively with the PTP method, according with these results and the congruence between morphology and GMYC analysis we keep the position of the species in the particular genera. However, in the cases where the over‐splitting revealed by GMYC is considered unjustifiable in many instances along the Stevardiinae tree, we adopt a conservative interpretation (*sensu* [100]) primarily based on results from the last two methods with fewer genetically defined entities.

In both GMYC and ABGD it was possible to obtain congruent delimitation results using the entire dataset (85.5%). However, in PTP when the outgroup was kept, the results showed a high undersplited delimitation, disagreement with morphological sources of evidence and the supports values were much lower. In contrast, when the outgroups were removed the congruence between the methods reaches the 65.4%, showing a best success in delimiting species.

On the contrary, the lower congruence in species delimitation between the PTP and GMYC methods, may be related to the unbalance represented in the data set. According to the literature [44] PTP method is more sensitive to this situation, this is, where the over-sampled species exhibit small within-species variation. Thus, under-sampling of rare species or over-sampled species with small intraspecific variation will compromise species delimitation. Additionally, the phylogenetic trees inferred on a single COI gene could also have some problems, such as incomplete lineage sorting, hybridization or in recent speciation events [92,96]. Similarly, when conflicting results occur in all methods, a detailed and careful inspection should be done [94]. Most of these incongruences were associated to certain species complexes clades In this context, different molecular methods provide a more wide view when defining PSH; for instance, scale of intra-or interspecific distances [94], since morphological variations are usually difficult to define in taxonomically complex groups.

Finally, regarding workflow performance, the results of the three methods (GMYC, PTP, ABGD) were consistent and concordant with species delimitation studies conducted on conflicting taxa [1,96,101]. Regardless of the set of parameters and phylogenetic models applied and the use of only single locus dataset, the results supports the validity of using DNA sequence data for species discrimination and identification of characins revealing potential misidentifications or junior synonyms. Likewise, species-level para-and polyphyly in DNA barcode gene trees could result in deep intraspecific divergence, which would help to detect presence of cryptic species.

### Taxonomic implications

For clade 1 (*Eretmobrycon*), the three molecular delimitation methods are congruent with molecular hypothesis [31] and previous results from morphology and sperm data [21,30] which also confirms the revalidation and inclusion of all *Bryconamericus* species distributed along the Pacific rivers in Colombia and Ecuador and Lower Central America in *Eretmobrycon*. The genus *Eretmobrycon* was described by Fink [86] from Rio Bayano based in morphological characters, however, Román-Valencia [85] considered *Eretmobrycon* as synonymous of *Bryconamericus* based only in morphometric analysis. From that point on, species described for the Pacific rivers were considered as *Bryconamericus* until Thomaz et al. [31].

Our results indicate there is a hidden biodiversity within *Eretmobrycon*, especially in morphospecies distributed in Ecuadorian and Colombian Pacific rivers (e.g. *E*. *peruanus* and *Eretmobrycon* sp.). OTUs considered as *E*. *emperador* reveal that this morphospecies is composed by at least six putative species clearly differentiated: *E*. *emperador* Dagua Group, *E*. *emperador* Atrato-Ichó Group, *E*. *emperador* Tuira-Yape Group, *E*. *emperador* San Juan Group, *E*. *emperador* Lower Central America Group A and *E*. *emperador* Lower Central America Group B (Fig 2, S1 Table). Although the analysis of delimitation of species show signs of putative species within this complex, we reject the OTUs recovered due to the incongruence among the methods. However, the low genetic differentiation observed between GMYC OTUs 35, 36, 37, 38, 39, 41, 42, 43, 44, 45, 46, 47 and 48 of *E*. *emperador* (S2 File) can be associated with population variation. These morphospecies are isolated in disconnected rivers of the Pacific ecoregion and it may be an indication that these species not yet reached the reciprocal monophyly.

This situation also is reflected in the taxonomy of *E*. *emperador* Eigenmann and Ogle 1907, which has experienced multiple changes since its initial description, including redescriptions and seven synonymizations with only two species considered valid *B*. *scopiferus* Eigenmann 1913 (Rio San Juan, Itsmina, Choco), and *B*. *terrabensis* Meek 1914 (Rio Grande de terraba, Costa Rica) and *B*. *zeteki* Hildebrand 1938 (a creek in El Valle, Pacific slope, Panamá) According to the results, this OTUs probably correspond to some of these species.

For the clade 2 (*Hemibrycon*), taxonomical inconsistencies are present yet. GMYC revealed only one error in the identification, in which a specimen of the species *H*. *metae* was listed as *Knodus*. However, studies to date aimed at defining the genus have failed to find exclusive synapomorphies in the external morphology and osteology, and this seems to be related to the conservative or primitive morphology of its representatives [102]. Due to this condition, species such *B*. *tolimae* Eigenmann 1913 and *B*. *caucanus* Eigenmann 1913 have generated debate regarding their possible taxonomic status within *Hemibrycon* [31,82].

An interesting result was the small genetic distances between *H*. *multiradiatus* and *Hemibrycon* aff. *raqueliae*. However, we consider them separate putative species because the integrative taxonomy analysis showed complete congruence between all methods, and the species are morphologically distinct and have restricted distributions in the Rio Atrato and small creeks and tributaries of the Rio Magdalena, respectively. The morphological reexamination of *Hemibrycon* aff. *raqueliae* represented an ambiguous identification due to the use of meristic characteristics that have some degree of congruence with the diagnosis of *H*. *raqueliae* [103], but based on our results and the comparison with topotypes (Rio Tasajo, Samaná, Caldas, Colombia), we recently described to *Hemibrycon* aff. *raqueliae* (Quebrada Batatas, Suarez, Tolima, Colombia) as one new species [104].

In the clade grouping individuals of *Hemibrycon plutarcoi* (*sensu* Thomaz et al. [31]) and *B*. *arilepis*, we observed contrasting results in the delimitation. Two molecular methods (ABGD and PTP) recovered *B*. *arilepis* and *H*. aff. *plutarcoi* as a single OTU, and a single specimen of *H*. aff. *plutarcoi* (GMYC OTU 71), was recovered as an independent OTU by two molecular methods (GMYC and ABGD). Therefore, there are two options: to consider that *B*. *arilepis* and *H*. *plutarcoi* constitute the same putative species or that they are different. Likewise, the GMYC OTU 71 may correspond to other species morphologically indistinguishable and closely related to species distributed in the Upper Magdalena Basin. We accept the last condition based on the morphological differences between them and the lack of reciprocal monophyly. However, a deeper morphological review is required for these species distributed in the Middle Magdalena Basin.

The complexity around *Bryconamericus* species is evident in the clade 3 with the greatest inconsistency between the morphological sorting and GMYC analysis. However, regardless of cataloging errors tissues listed above, clades 3a and 3b are cis-Andean species belonging to *Bryconamericus*, *Piabarchus*, *Piabina* and *Diapoma*. Rigorous morphological examination of the type species *B*. *exodon* (collected in the type locality), demonstrates that species boundaries are unclear but that there is also suspicious sorting and position in the GMYC tree of the *Piabarchus* species, since *P*. *analis* corresponds to the type species and that *P*. *stramineus* and *P*. *thomasi* recently were reassigned from *Bryconamericus* to *Piabina* [31]. A re-description and osteological study of *Bryconamericus exodon* [68] showed that *B*. *stramineus* is the species morphologically more similar, and their analysis did not reveal any clear apomorphic state for *B*. *exodon* and all other species of the genus examined, including *B*. *turiuba*. This species is found in the same GMYC group with *B*. *exodon*. In this context, our analyzes suggest that *B*. *turiuba* is a junior synonym of *B*. *exodon*. This proposal also is supported in other studies [67] that recognized three groups of *Bryconamericus* species from southern South America based mainly in the position and form of the maxillary teeth. Also taxonomic uncertainty about the type species has become so important that some studies have included species listed as ‘affinis’ in their analysis [105]. This study found multiple NORs (Nucleolar organizer regions) in *Bryconamericus* aff. *exodon* from Tibagi, and therefore, our findings provide insights on the limits of the type species of *Bryconamericus* regarding their true taxonomic status. This situation raises doubt as to whether the species used in some previous phylogenetic studies [31,106], as *B*. *exodon*, truly corresponds to the correct entity.

In the same way, ABGD and PTP methods consider to *B*. *andresoi* and *B*. *galvisi* as a unique putative species, as these two species are morphologically distinct [85,107] with restricted distribution in the upper Rio Patía (Pacific region) and Rio Putumayo (Amazonas Basin), respectively. These rivers originated in the Colombian Massif, so it is likely that these species did not reach the reciprocal monophyletic due to the young formation during the formation of this basins with a short divergence time. Similar studies within Loricariidae [6] considered the morphologic distinction sufficient to validate two species independently of low genetic divergence, and therefore, we accept the delimitation of these species, which also occupy different habitat types and probably have specific morphological adaptations of each environment. Our results also call into question the validity of species such as *B*. *caldasi* and *B*. *huilae*, which are recovered as single OTUs with *Hemibrycon* aff. *caucanus* and *H*. *tolimae* respectively. This result was predictable due to diagnoses ambiguity with variables characters with little phylogenetic support (e.g. the number of predorsal scales, a peduncular red spot) proposed by Román-Valencia et al. [63,108,109]. In addition, our morphological review found that *B*. *huilae* (*sensu* Román-Valencia [107]) has supraneurals and the distinction from *B*. *tolimae* turns out to be artificial because it relies on characteristics with little or no taxonomic value.

Given the above considerations, we agree with García et al. [104] in to propose that some species currently consider in *Bryconamericus* must be reassigned to the genus *Hemibrycon*; based on the high degree of congruence of molecular methods and weak taxonomic diagnosis in the original descriptions of species; other species could also be considered synonyms (see Table 2).

After a morphological re-examination in the Clade 3b, *B*. *iheringii* was the species with more re-assignments, resulting in polyphyly and the greatest number of inconsistencies. This situation can be due to great morphological similarity with several other described species, such as *B*. *eigenmanni*, *B*. *rubropictus B*. *sylvicola*, *B*. *ikaa* and *Piabina thomasi*. One of the groups recognized by Pezzi da Silva [67] is the *B*. *iheringii* group, which includes all other species from southern South America (*B*. *patriciae*, *B*. *ecai* and *B*. *ikaa*). The complex taxonomic status of these species distributed in the Paraná River Basin is demonstrated by the morphological analysis that has not revealed any unequivocal apomorphic state [68] and the great morphological similarity in sympatric species of *Bryconamericus* and related genera. The problem is magnified in species with a wide geographical distribution range as *B*. *iheringii*, which occurs in different environments of the La Plata River basin [110,111]. However, the great chromosomal variability observed in *Bryconamericus* species of the Rio Paraná suggest that divergent karyotypic evolution may have occurred [112] with distinct evolutionary forces acting on the diversity of rDNA sequences in the genome [113], which may explain the taxonomic complexity observed in these species.

As occurs in *Eretmobrycon*, *Hemibrycon* and *Bryconamericus*, *Knodus* have been traditionally and arbitrarily diagnosed using criteria based on the number of teeth on the maxilla and the extension of scales over the caudal-fin rays, which generates uncertainty on the number of valid species in the genus. However, this study shows a clear delimitation of the genera (Clade 4) with exclusively widely distributed cis-Andean species, except *Knodus meridae*. Although the monophyly of this genus in previous phylogenetic analysis has been strongly rejected by the molecular data (i.e. Thomaz et al. [31]), it could be due to erroneous identifications of the included species and poor taxonomic sampling. In our study, many of the species listed in the collections as *Bryconamericus* correspond to misidentified *Knodus* species (OTUs GMYC 129, 140, 150, 151, 153,157), highlighting the difficulties in the delimitation of the two genera using only external morphological characteristics.

According with our results, the caudal scalation characteristic of *Knodus* observed by Román-Valencia et al. [77] is not taxonomically or phylogenetically useful, so we propose that *B*. *macarena*, *B*. *cismontanus* and *B*. *diaphanus* should be included in *Knodus*. Our results are consistent with other studies findings [28] and propose seven new potential species for the Orinoco and Guaviare Basins (Fig 1).

Misidentification of *Othonocheirodus* as *Creagrutus* in clade 4 may be due to the similarity in the position of the mouth in and the number teeth in the inner premaxillary row [114]. It is important to include *Othonocheirodus* species in future studies to define the limits of the genus and elucidate their phylogenetic relationships.

## Conclusions

Methods based in one loci analysis (e.g. mtDNA) are useful as a first approach in integrative taxonomy until multilocus or genomics approaches become feasible for broad surveys of entire clades or faunas and accessible to a representative number of taxonomists in developing countries. Our results highlight the importance of the species delimitation through the integrative framework that could strength the taxonomy and classification of Characidae, crucial for studies in phylogeny, phylogeography, ecology, conservation and biogeography of Neotropical freshwater fishes. We consider that difficulties to recognize the boundaries at the species or genus levels in *Bryconamericus*, *Eretmobrycon*, *Knodus*, and *Hemibrycon* can be overcome through exploratory analyzes delimitation that present a putative species as guide and comparing with morphology and other methods.

## Supporting information

S1 Table Samples data summary and taxonomic update. (XLSX)

S1 Fig Test of substitution saturation. (TIF)

S2 Fig NJ Kimura2P Tree. (PDF)

S3 Fig Maximum Likelihood Tree. (PDF)

S4 Fig Lineages-through-time plot for GMYC threshold. The vertical red line represents the timing of the earliest coalescent event.
(PDF)

S5 Fig Barcoding GAP. (JPG)

S6 Fig First run in PTP Maximum likelihood solution, without removing the sequences that were allocated outside of Stevardiinae in the GMYC analysis. (PDF)

S7 Fig Second run in PTP Maximum likelihood solution, removing the sequences that were allocated outside of Stevardiinae in the GMYC analysis. (PDF)

S8 Fig ABGD Results, with the number of partitions obtained in each prior threshold for COI. (JPG)

S1 File Maximum Likelihood partition with GMYC analysis. (PDF)

S2 File Genetic distances between GMYC OTUs less than 2%. Standard error estimate(s) are shown in the last column. Analyses were conducted using the Kimura 2-parameter model.
(XLSX)

S3 File Maximum Likelihood partition with PTP analysis. The sequences allocated outside of Stevardiinae in the GMYC analysis, were not removed.
(TXT)

S4 File Maximum Likelihood partition with PTP analysis. The sequences allocated outside of Stevardiinae in the GMYC analysis, were removed.
(DOCX)
