# Supplementary figures and images for: Species delimitation of neotropical Characins (Stevardiinae): Implications for taxonomy of complex groups
Source: PLoS One. 2019 Jun 5;14(6):e0216786. doi: 10.1371/journal.pone.0216786 (PMC6550444; doi:10.1371/journal.pone.0216786)

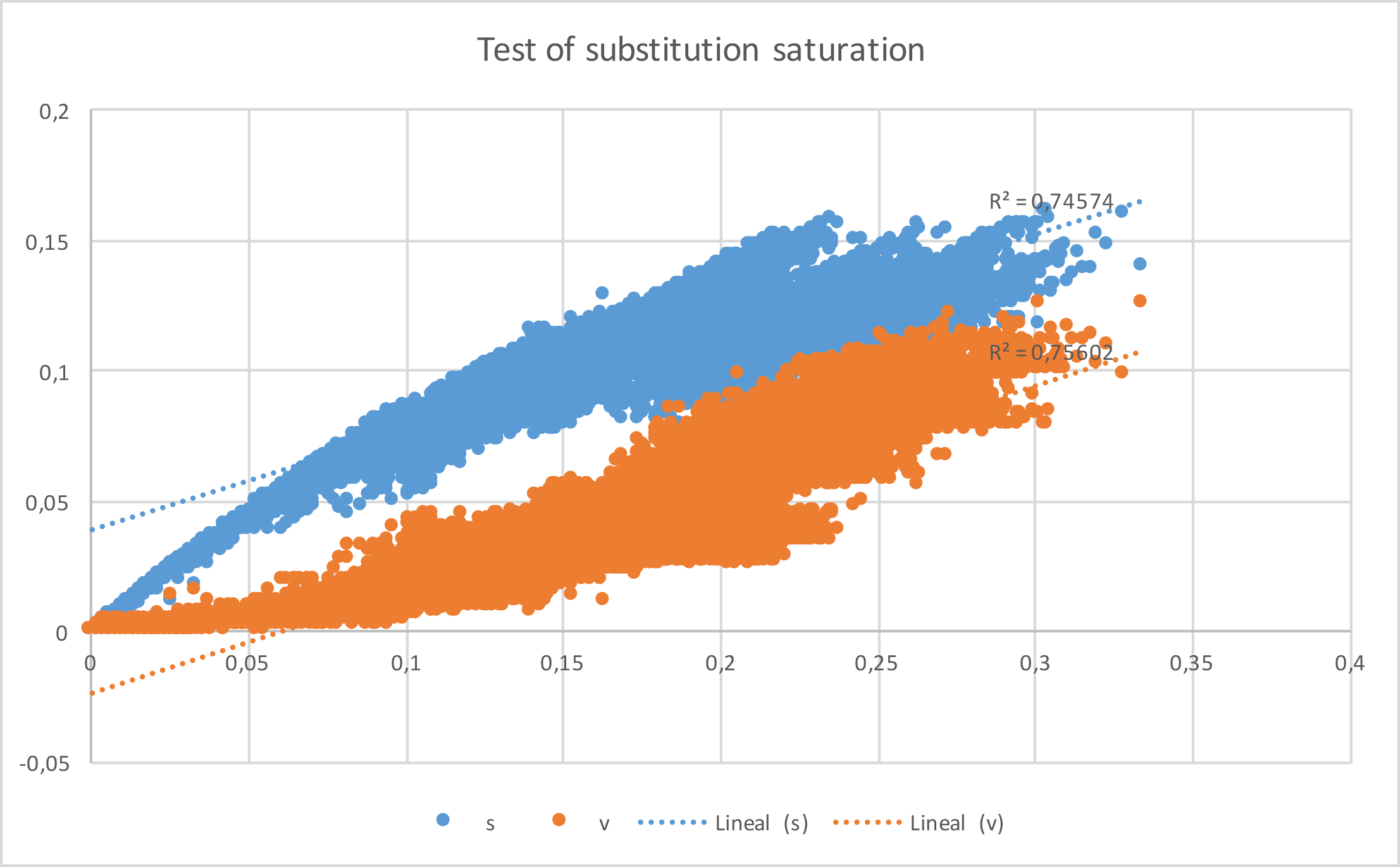

Supplement: S1 Fig — (TIF) [file pone.0216786.s002.tif]

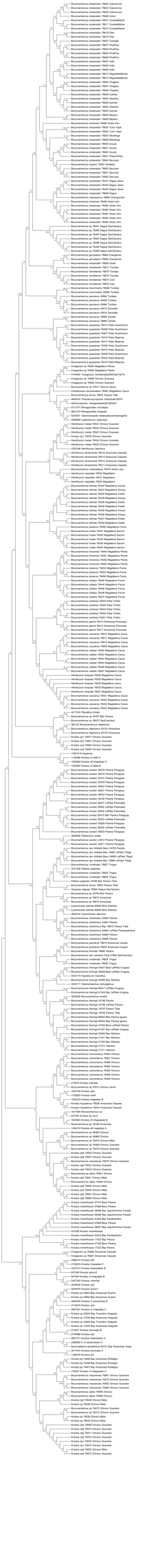

Supplement: S2 Fig — (PDF) [file pone.0216786.s003.pdf]

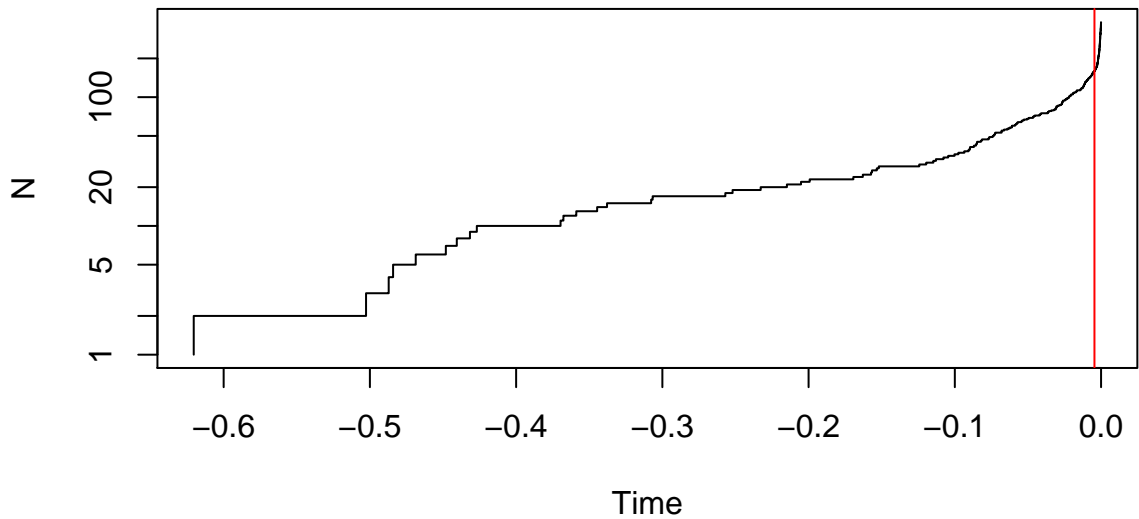

Supplement: S4 Fig — The vertical red line represents the timing of the earliest coalescent event. (PDF) [file pone.0216786.s005.pdf]

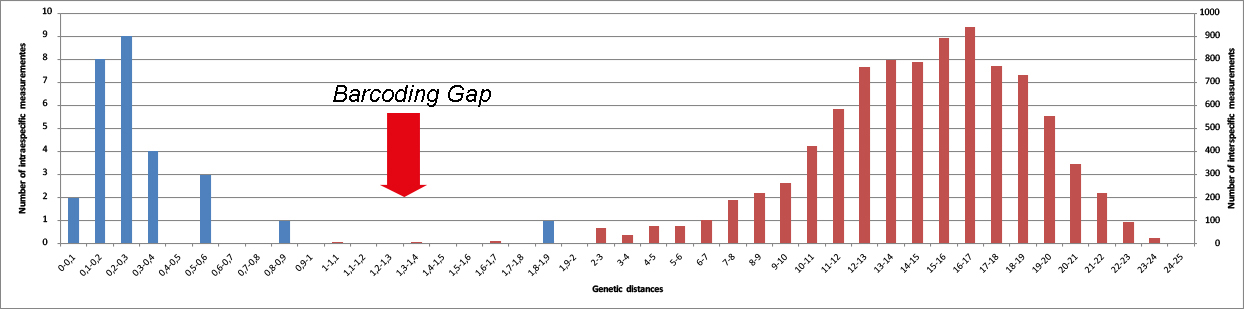

Supplement: S5 Fig — (JPG) [file pone.0216786.s006.jpg]

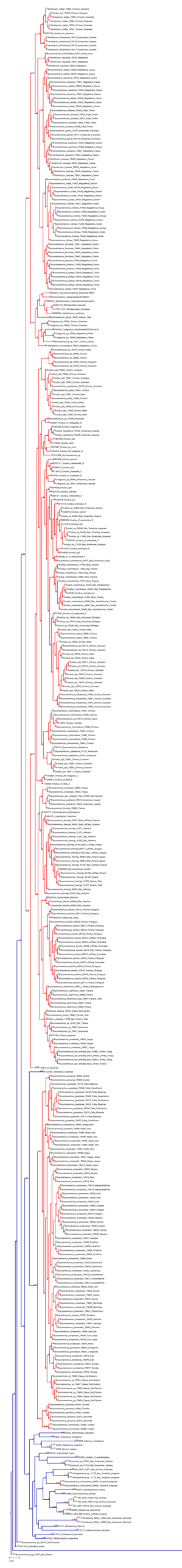

Supplement: S6 Fig — (PDF) [file pone.0216786.s007.pdf]

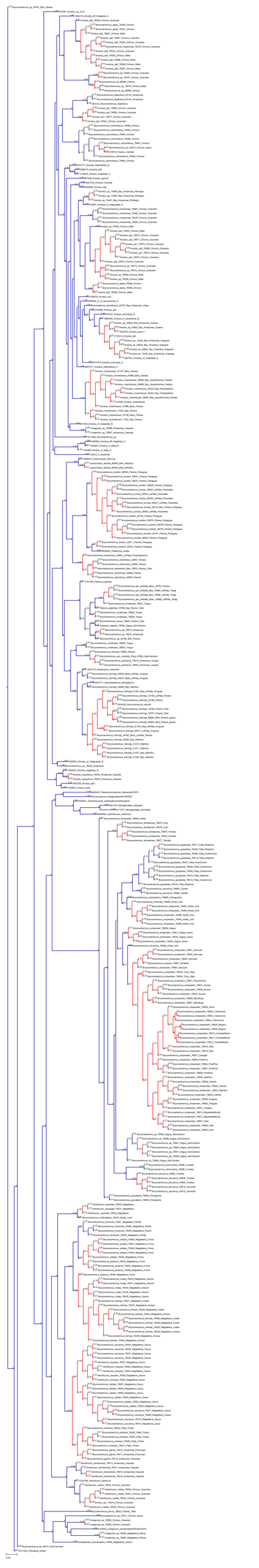

Supplement: S7 Fig — (PDF) [file pone.0216786.s008.pdf]

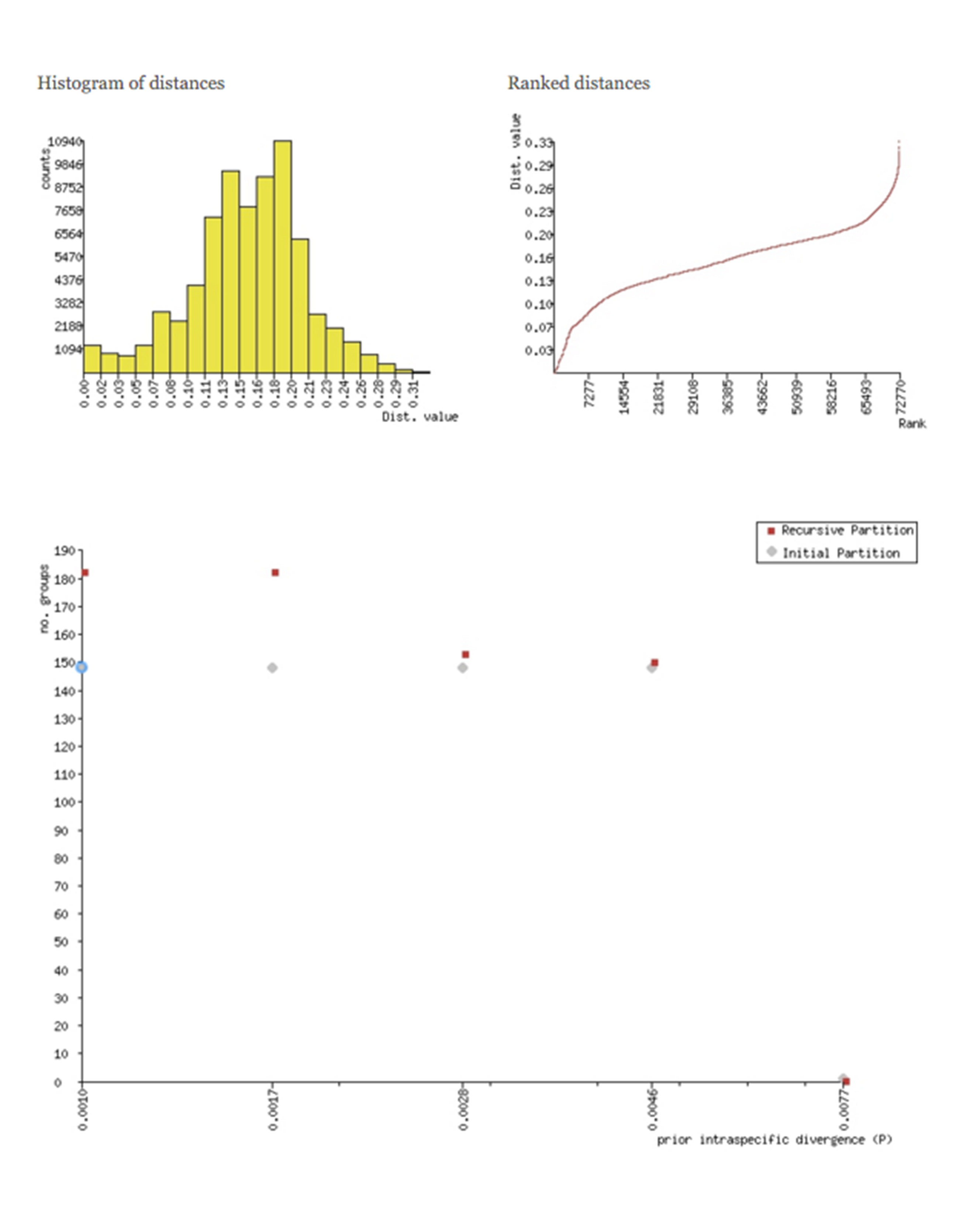

Supplement: S8 Fig — (JPG) [file pone.0216786.s009.jpg]
