# Supplementary material for: Species delimitation of neotropical Characins (Stevardiinae): Implications for taxonomy of complex groups
Source: PLoS One. 2019 Jun 5;14(6):e0216786. doi: 10.1371/journal.pone.0216786 (PMC6550444; doi:10.1371/journal.pone.0216786)
Supplement: S3 Fig — (PDF) [file pone.0216786.s004.pdf]

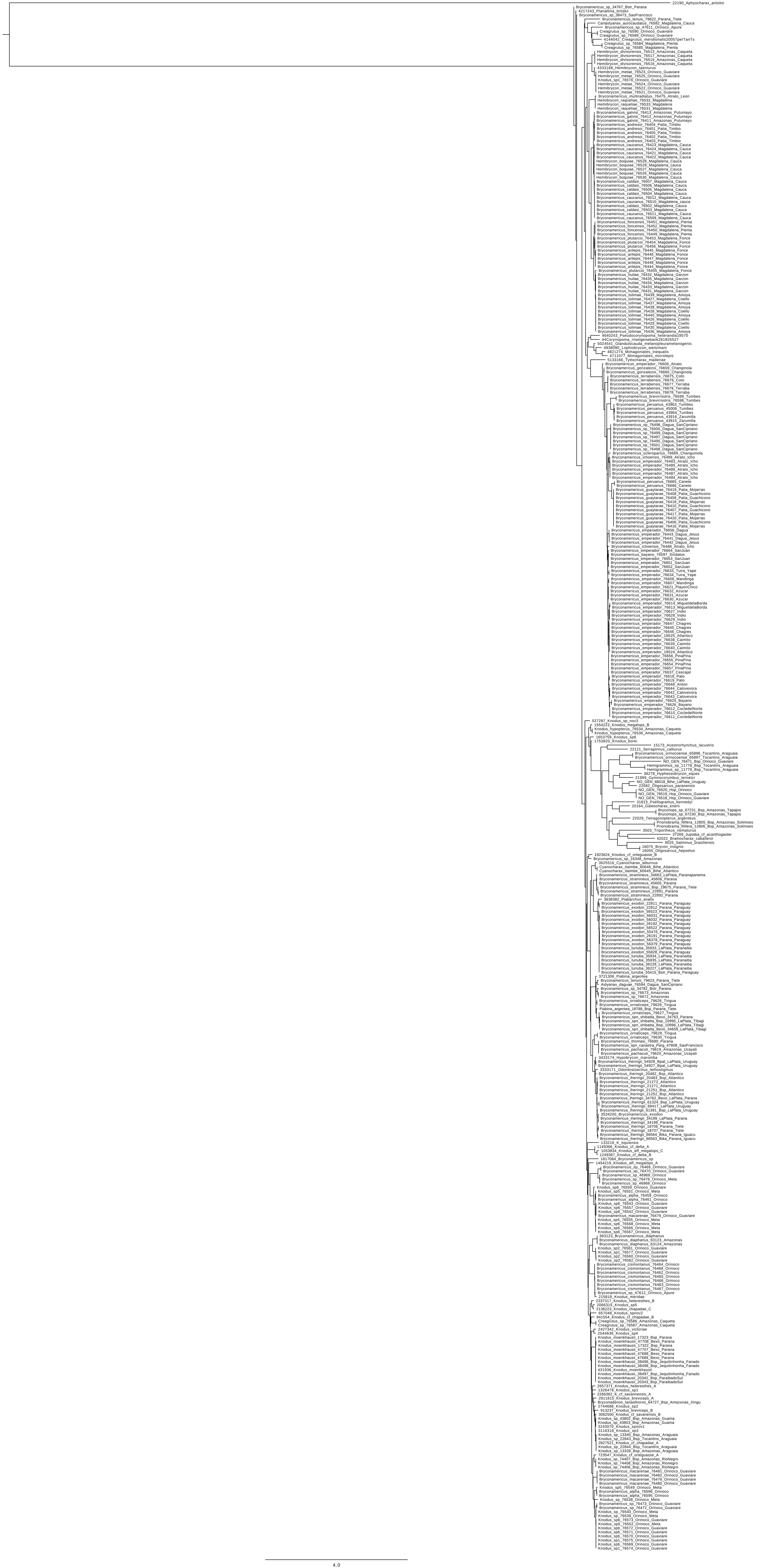
4.0
