# Supplementary material for: Species delimitation of neotropical Characins (Stevardiinae): Implications for taxonomy of complex groups
Source: PLoS One. 2019 Jun 5;14(6):e0216786. doi: 10.1371/journal.pone.0216786 (PMC6550444; doi:10.1371/journal.pone.0216786)
Supplement: S1 File — (PDF) [file pone.0216786.s010.pdf]

Max likilhood partition
Species 1 (support = 1.000)
22190_Aphyocharax_anisitsi

Species 2 (support = 0.998)

Prionobrama_filifera_12806_Bsp_Amazonas_Solimoes,Prionobrama_filifera_12805_Bsp_Amazonas_Solimoes

Species 3 (support = 1.000)
22029_Tetragonopterus_argenteus

Species 4 (support = 1.000)
3503_Triportheus_nematurus

Species 5 (support = 0.994)

Bryconamericus_orinocoense_65897_Tocantins_Araguaia,Bryconamericus_orinocoense_65896_Tocantins_Araguaia

Species 6 (support = 0.999)

Bryconops_sp_67230_Bsp_Amazonas_Tapajos,Bryconops_sp_67231_Bsp_Amazonas_Tapajos

Species 7 (support = 1.000)
37269_Jupiaba_cf_acanthogaster

Species 8 (support = 1.000)
42022_Bramocharax_caballeroi

Species 9 (support = 1.000)
51-33166_Tyttocharax_madeirae

Species 10 (support = 1.000)
22121_Serrapinnus_calliurus

Species 11 (support = 1.000)
15173_Acestrorhynchus_lacustris

Species 12 (support = 1.000)
16055_Oligosarcus_hepsetus,16075_Brycon_insignis

Species 13 (support = 1.000)
9025_Salminus_brasiliensis

Species 14 (support = 0.958)

Hemigrammus_sp_11779_Bsp_Tocantins_Araguaia,Hemigrammus_sp_11778_Bsp_Tocantins_Araguaia

Species 15 (support = 1.000)
NO_GEN_76471_Bsp_Orinoco_Guaviare

Species 16 (support = 1.000)
21989_Gymnocorymbus_ternetzi

Species 17 (support = 1.000)
36278_Hyphessobrycon_eques

Species 18 (support = 1.000)
31813_Psellogramus_kennedyi

Species 19 (support = 1.000)
20164_Galeocharax_knerii

Species 20 (support = 0.998)

NO_GEN_76518_Hsp_Orinoco_Guaviare,NO_GEN_76520_Hsp_Orinoco,NO_GEN_7651
9_Hsp_Orinoco_Guaviare

Species 21 (support = 1.000)
Bryconamericus_tenuis_79622_Parana_Tiete

Species 22 (support = 1.000)
44-Corynopoma_riisei

Species 23 (support = 1.000)
46-40243_Pseudocorynopoma_heterandia

Species 24 (support = 1.000)
Carlastyanax_aurocaudatus_76582_Magdalena_Cauca

Species 25 (support = 1.000)
'1-33216_K._tiquiensis'

Species 26 (support = 1.000)
18-17084_Bryconamericus_sp

Species 27 (support = 1.000)
41-44042_Creagrutus_meridionalis

Species 28 (support = 1.000)
Bryconamericus_sp_47611_Orinoco_Apure

Species 29 (support = 1.000)
22582_Oligosarcus_paranensis

Species 30 (support = 1.000)
NO_GEN_68418_Bihe_LaPlata_Uruguay

Species 31 (support = 0.970)

Creagrutus_sp_76584_Magdalena_Pienta,Creagrutus_sp_76585_Magdalena_Pienta

Species 32 (support = 1.000)

Creagrutus_sp_76589_Orinoco_Guaviare,Creagrutus_sp_76590_Orinoco_Guaviare

Species 33 (support = 1.000)
'6-57048_Knodus_sp.nov.2'

Species 34 (support = 1.000)
'5-27297_Knodus_sp._nov3'

Species 35 (support = 1.000)
'14-54219_Knodus_aff._megalops_A'

Species 36 (support = 1.000)
'19-23624_Knodus_cf._orteguasse_B'

Species 37 (support = 1.000)
Bryconamericus_sp_16348_Amazonas

Species 38 (support = 1.000)
'8-41554_Knodus_cf._chapadae_B'

Species 39 (support = 0.996)

Knodus_moenkhausii_38497_Bsp_Jequitinhonha_Fanado,Knodus_moenkhausii_38498_Bsp_Jequitinhonha_Fanado,Knodus_moenkhausii_17323_Bsp_Parana,Knodus_moenkhausii_38496_Bsp_Jequitinhonha_Fanado,Knodus_moenkhausii_20342_Bsp_ParaibadoSul,Knodus_moenkhausii_47689_Bexo_Parana,Knodus_moenkhausii_20343_Bsp_ParaibadoSul,Knodus_moenkhausii_47688_Bexo_Parana,Knodus_moenkhausii_47707_Bexo_Parana,
4-31936_Knodus_moenkhausii,Knodus_moenkhausii_17322_Bsp_Parana,Knodus_moenkhausii_47708_Bexo_Parana

Species 40 (support = 0.515)

Bryconamericus_peruanus_43964_Tumbes,Bryconamericus_peruanus_45006_Tumbes,Bryconamericus_peruanus_43963_Tumbes,Bryconamericus_peruanus_43916_Zarumilla,Bryconamericus_peruanus_43915_Zarumilla

Species 41 (support = 0.999)

Bryconamericus_brevirrostris_76599_Tumbes,Bryconamericus_brevirrostris_76598_Tumbes

Species 42 (support = 0.988)

Bryconamericus_pachacuti_79620_Amazonas_Ucayali,Bryconamericus_pachacuti_79619_Amazonas_Ucayali,Bryconamericus_thomasi_76680_Parana,Bryconamericus_spn_canastra_Parg_47808_SaoFrancisco

Species 43 (support = 0.996)

Bryconamericus_ornaticeps_79629_Tingua,Bryconamericus_ornaticeps_79630_Tingua

Species 44 (support = 1.000)
13-26478_Knodus_sp1

Species 45 (support = 0.938)

Knodus_sp2_76562_Orinoco_Guaviare,Knodus_sp2_76560_Orinoco_Guaviare,Knodus_sp1_76577_Orinoco_Guaviare,Knodus_sp2_76561_Orinoco_Guaviare

Species 46 (support = 0.992)

3-63123_Bryconamericus_diaphanus,Bryconamericus_diaphanus_63123_Amazonas,Bryconamericus_diaphanus_63124_Amazonas

Species 47 (support = 1.000)
26-57371_Knodus_heteresthes_A

Species 48 (support = 0.992)

Hemibrycon_divisorensis_76515_Amazonas_Caqueta,Hemibrycon_divisorensis_76513_Amazonas_Caqueta,Hemibrycon_divisorensis_76516_Amazonas_Caqueta,Hemibrycon_divisorensis_76517_Amazonas_Caqueta

Species 49 (support = 0.999)

Bryconamericus_macarenae_76480_Orinoco_Guaviare,Bryconamericus_macarenae_76481_Orinoco_Guaviare,Bryconamericus_macarenae_76482_Orinoco_Guaviare,Bryconamericus_macarenae_76478_Orinoco_Guaviare

Species 50 (support = 1.000)
'22-66362_K._cf._savannensis_A'

Species 51 (support = 1.000)
47-11077_Mimagoniates_microlepis

Species 52 (support = 1.000)
48-21274_Mimagoniates_inequalis

Species 53 (support = 0.979)

Bryconamericus_sp_76476_Orinoco_Meta,Bryconamericus_sp_46968_Orinoco,Bryconamericus_sp_46969_Orinoco

Species 54 (support = 0.509)

Bryconamericus_sp_76469_Orinoco_Guaviare,Bryconamericus_sp_76470_Orinoco_Guaviare

Species 55 (support = 1.000)
49-38090_Lophiobrycon_weitzmani

Species 56 (support = 1.000)
50-24541_Glandulocauda_melanopleura

Species 57 (support = 1.000)
23-37317_Knodus_heteresthes_B

Species 58 (support = 0.996)

Bryconamericus_sp_76497_Dagua_SanCipriano,Bryconamericus_sp_76500_Dagua_SanCipriano,Bryconamericus_sp_76496_Dagua_SanCipriano,Bryconamericus_sp_76495_Dagua_SanCipriano,Bryconamericus_sp_76498_Dagua_SanCipriano,Bryconamericus_sp_76499_Dagua_SanCipriano,Bryconamericus_sp_76501_Dagua_SanCipriano

Species 59 (support = 1.000)
29-11815_Knodus_breviceps_A

Species 60 (support = 0.864)

Bryconamericus_alpha_76595_Orinoco,Bryconamericus_alpha_76596_Orinoco,Knodus_sp5_76549_Orinoco_Meta

Species 61 (support = 1.000)
Knodus_sp_76538_Orinoco_Meta

Species 62 (support = 1.000)
Bryconadenos_tanaothoros_64727_Bsp_Amazonas_Xingu

Species 63 (support = 1.000)
Bryconamericus_iheringii_20482_Bsp_Atlantico

Species 64 (support = 1.000)
33-33171_Odontostoechus_lethostigmus

Species 65 (support = 0.924)

Bryconamericus_guaytarae_76409_Patia_Guachicono,Bryconamericus_guaytarae_76419_Patia_Mojarras,Bryconamericus_guaytarae_76420_Patia_Mojarras,

Bryconamericus_guaytarae_76410_Patia_Guachicono,Bryconamericus_guaytarae_76408_Patia_Guachicono,Bryconamericus_guaytarae_76417_Patia_Mojarras,Bryconamericus_guaytarae_76418_Patia_Mojarras,Bryconamericus_guaytarae_76416_Patia_Mojarras,Bryconamericus_guaytarae_76407_Patia_Guachicono,Bryconamericus_guaytarae_76406_Patia_Guachicono

Species 66 (support = 0.999)

Bryconamericus_peruanus_76665_Canete,Bryconamericus_peruanus_76666_Canete

Species 67 (support = 0.995)

Bryconamericus_emperador_76443_Dagua_Jesus,Bryconamericus_emperador_76441_Dagua_Jesus,Bryconamericus_emperador_76442_Dagua_Jesus,Bryconamericus_emperador_76658_Dagua

Species 68 (support = 1.000)
16-53759_Knodus_sp6

Species 69 (support = 1.000)
17-53820_Knodus_borki

Species 70 (support = 1.000)
27-44688_Knodus_sp2

Species 71 (support = 0.980)

Knodus_sp_74407_Bsp_Amazonas_RioNegro,Knodus_sp_74408_Bsp_Amazonas_Rionegro,Knodus_sp_74406_Bsp_Amazonas_RioNegro

Species 72 (support = 1.000)
'7-23547_Knodus_cf._orteguasse_A'

Species 73 (support = 1.000)
Bryconamericus_sp_38473_SaoFrancisco

Species 74 (support = 0.999)

Hemibrycon_metae_76525_Orinoco_Guaviare,Hemibrycon_metae_76522_Orinoco_Guaviare,Hemibrycon_metae_76521_Orinoco_Guaviare,Knodus_sp1_76578_Orinoco_Guaviare,Hemibrycon_metae_76524_Orinoco_Guaviare,Hemibrycon_metae_76523_Orinoco_Guaviare

Species 75 (support = 1.000)
43-33168_Hemibrycon_taeniurus

Species 76 (support = 1.000)
9-13237_Knodus_breviceps_B

Species 77 (support = 1.000)
'11-49366_Knodus_cf._delta_A'

Species 78 (support = 1.000)
38-38382_Piabarchus_analis

Species 79 (support = 0.928)

Bryconamericus_exodon_56379_Parana_Paraguay,Bryconamericus_exodon_5637
8_Parana_Paraguay,Bryconamericus_exodon_26191_Parana_Paraguay,Bryconam
ericus_exodon_55476_Parana_Paraguay,Bryconamericus_exodon_56522_Parana
_Paraguay,Bryconamericus_turiuba_36227_LaPlata_Paranaiba,Bryconamericu
s_exodon_55828_Parana_Paraguay,Bryconamericus_exodon_26192_Parana_Para
guay,Bryconamericus_turiuba_35935_LaPlata_Paranaiba,Bryconamericus_tur
iuba_36228_LaPlata_Paranaiba,Bryconamericus_turiuba_35933_LaPlata_Para
naiba,Bryconamericus_turiuba_55415_Bstr_Parana_Paraguay,Bryconamericus
_exodon_56032_Parana_Paraguay,Bryconamericus_exodon_56031_Parana_Parag
uay,Bryconamericus_turiuba_35934_LaPlata_Paranaiba,Bryconamericus_exod
on_56523_Parana_Paraguay

Species 80 (support = 0.994)

Cyanocharax_itaimbe_60646_Bihe_Atlantico,Cyanocharax_itaimbe_60645_Bih
e_Atlantico

Species 81 (support = 1.000)
36-25516_Cyanocharax_alburnus

Species 82 (support = 1.000)
20-66315_Knodus_sp5

Species 83 (support = 1.000)
21-36223_Knodus_chapadae_C

Species 84 (support = 0.996)

Bryconamericus_terrabensis_76675_Coto,Bryconamericus_terrabensis_76676
_Coto,Bryconamericus_terrabensis_76679_Terraba,Bryconamericus_terraben
sis_76678_Terraba,Bryconamericus_terrabensis_76677_Terraba

Species 85 (support = 0.505)

Knodus_hypopterus_76536_Amazonas_Caqueta,Knodus_hypopterus_76534_Amazo
nas_Caqueta

Species 86 (support = 1.000)
15-54223_Knodus_megalops_B

Species 87 (support = 1.000)
Knodus_sp6_76559_Orinoco_Guaviare

Species 88 (support = 1.000)
25-44636_Knodus_sp4

Species 89 (support = 1.000)
24-27342_Knodus_victoriae

Species 90 (support = 0.997)

Bryconamericus_iheringii_54927_Bpat_LaPlata_Uruguay,Bryconamericus_iheringii_54928_Bpat_LaPlata_Uruguay

Species 91 (support = 1.000)
34-33174_Hypobrycon_maromba

Species 92 (support = 1.000)
'30-62500_Knodus_cf._savanensis_B'

Species 93 (support = 1.000)
Bryconamericus_emperador_76606_Atrato

Species 94 (support = 0.953)

Bryconamericus_emperador_76483_Atrato_Icho,Bryconamericus_emperador_76487_Atrato_Icho,Bryconamericus_emperador_76484_Atrato_Icho,Bryconamericus_emperador_76486_Atrato_Icho,Bryconamericus_emperador_76485_Atrato_Icho,Bryconamericus_ichoensis_76489_Atrato_Icho

Species 95 (support = 1.000)
Bryconamericus_scleroparius_76669_Changuinola

Species 96 (support = 0.998)

Bryconamericus_iheringii_68417_LaPlata_Uruguay,Bryconamericus_iheringii_61324_Bsp_LaPlata_Uruguay

Species 97 (support = 1.000)
Bryconamericus_ichoensis_76488_Atrato_Icho

Species 98 (support = 1.000)
42-17243_Planaltina_britskii

Species 99 (support = 1.000)
Bryconamericus_sp_34767_Bstr_Parana

Species 100 (support = 1.000)
Bryconamericus_sp_47612_Orinoco_Apure

Species 101 (support = 0.952)

Hemibrycon_raqueliae_76531_Magdalena,Hemibrycon_raqueliae_76532_Magdalena,Hemibrycon_raqueliae_76533_Magdalena

Species 102 (support = 1.000)
Bryconamericus_multiradiatus_76475_Atrato_Leon

Species 103 (support = 1.000)
37-21306_Piabina_argentea

Species 104 (support = 1.000)

Bryconamericus_ornaticeps_79626_Tingua,Bryconamericus_ornaticeps_79628_Tingua

Species 105 (support = 1.000)
Piabina_argentea_18788_Bsp_Parana_Tiete

Species 106 (support = 0.991)

Bryconamericus_sp_76472_Orinoco_Guaviare,Bryconamericus_sp_76473_Orinoco_Guaviare

Species 107 (support = 0.992)

Bryconamericus_emperador_76633_Tuira_Yape,Bryconamericus_emperador_76634_Tuira_Yape

Species 108 (support = 0.986)

Bryconamericus_emperador_76618_Pato,Bryconamericus_emperador_76619_Pato

Species 109 (support = 0.998)

Bryconamericus_emperador_76625_Bayano,Bryconamericus_emperador_76626_Bayano

Species 110 (support = 0.546)

Bryconamericus_emperador_76640_Caimito,Bryconamericus_emperador_18525_Atlantico,Bryconamericus_emperador_76639_Caimito,Bryconamericus_emperador_18524_Atlantico,Bryconamericus_emperador_76638_Caimito,Bryconamericus_emperador_76646_Chagres,Bryconamericus_emperador_76647_Chagres,Bryconamericus_emperador_76645_Chagres,Bryconamericus_emperador_76613_MigueldelaBorda,Bryconamericus_emperador_76614_MigueldelaBorda,Bryconamericus_emperador_76629_Indio,Bryconamericus_emperador_76627_Indio,Bryconamericus_emperador_76628_Indio,Bryconamericus_emperador_76657_PinaPina,Bryconamericus_emperador_76656_PinaPina,Bryconamericus_emperador_76637_Cascajal,Bryconamericus_emperador_76655_PinaPina,Bryconamericus_emperador_76654_PinaPina

Species 111 (support = 0.965)

Bryconamericus_emperador_76632_Azucar,Bryconamericus_emperador_76630_Azucar,Bryconamericus_emperador_76631_Azucar,Bryconamericus_emperador_76621_PlayonChico

Species 112 (support = 0.933)

Bryconamericus_emperador_76607_Mandinga,Bryconamericus_emperador_76608_Mandinga

Species 113 (support = 0.988)

Knodus_sp_22843_Bsp_Tocantins_Araguaia,Knodus_sp_13339_Bsp_Amazonas_Araguaia,Knodus_sp_13340_Bsp_Amazonas_Araguaia,'28-27521_Knodus_cf._chapadae_A',Knodus_sp_22844_Bsp_Tocantins_Araguaia

Species 114 (support = 1.000)
Bryconamericus_emperador_76648_Anton

Species 115 (support = 0.998)

Bryconamericus_bayano_76597_Sindatos,Bryconamericus_emperador_76651_SanJuan,Bryconamericus_emperador_76653_SanJuan,Bryconamericus_emperador_76652_SanJuan

Species 116 (support = 1.000)
Bryconamericus_emperador_76664_SanJuan

Species 117 (support = 0.826)

Bryconamericus_arilepis_76447_Magdalena_Fonce,Bryconamericus_arilepis_76445_Magdalena_Fonce,Bryconamericus_arilepis_76444_Magdalena_Fonce,Bryconamericus_arilepis_76446_Magdalena_Fonce,Bryconamericus_arilepis_76448_Magdalena_Fonce,Bryconamericus_plutarcoi_76454_Magdalena_Fonce,Bryconamericus_plutarcoi_76456_Magdalena_Fonce,Bryconamericus_plutarcoi_76453_Magdalena_Fonce

Species 118 (support = 0.996)

Bryconamericus_foncensis_76452_Magdalena_Pienta,Bryconamericus_foncensis_76451_Magdalena_Pienta,Bryconamericus_foncensis_76450_Magdalena_Pienta,Bryconamericus_foncensis_76449_Magdalena_Pienta

Species 119 (support = 0.998)

Bryconamericus_iheringii_66563_Bika_Parana_Iguacu,Bryconamericus_iheringii_66564_Bika_Parana_Iguacu

Species 120 (support = 0.986)
Knodus_sp_43803_Bsp_Amazonas_Guama,'32-43070_Knodus_sp.nov.
1',Knodus_sp_43802_Bsp_Amazonas_Guama

Species 121 (support = 0.999)
31-16318_Knodus_sp3

Species 122 (support = 0.999)

Bryconamericus_emperador_76612_CocledelNorte,Bryconamericus_emperador_
76610_CocledelNorte,Bryconamericus_emperador_76611_CocledelNorte

Species 123 (support = 0.998)

Bryconamericus_emperador_76644_Calovevora,Bryconamericus_emperador_766
43_Calovevora,Bryconamericus_emperador_76642_Calovevora

Species 124 (support = 0.981)

Bryconamericus_iheringii_21271_Atlantico,Bryconamericus_iheringii_2127
2_Atlantico,Bryconamericus_iheringii_21252_Bsp_Atlantico,Bryconamericu
s_iheringii_21251_Bsp_Atlantico,Bryconamericus_iheringii_20483_Bsp_Atl
antico

Species 125 (support = 0.957)

Bryconamericus_iheringii_34198_Parana,Bryconamericus_iheringii_34199_L
aPlata_Parana,35-34200_Bryconamericus_exodon

Species 126 (support = 0.989)

Bryconamericus_iheringii_18707_Parana_Tiete,Bryconamericus_iheringii_1
8706_Parana_Tiete

Species 127 (support = 0.802)

Bryconamericus_caucanus_76509_Magdalena_Cauca,Bryconamericus_caldasi_7
6503_Magdalena_Cauca,Bryconamericus_caldasi_76505_Magdalena_Cauca,Bryc
onamericus_caucanus_76510_Magdalena_cauca,Bryconamericus_caucanus_7651
2_Magdalena_Cauca,Bryconamericus_caldasi_76502_Magdalena_Cauca,Brycona
mericus_caucanus_76511_Magdalena_Cauca,Bryconamericus_caldasi_76504_Ma
gdalena_Cauca,Bryconamericus_caldasi_76506_Magdalena_Cauca,Bryconameri
cus_caldasi_76507_Magdalena_Cauca,Hemibrycon_boquiae_76527_Magdalena_C
auca,Hemibrycon_boquiae_76528_Magdalena_Cauca,Hemibrycon_boquiae_76530
_Magdalena_Cauca,Hemibrycon_boquiae_76526_Magdalena_Cauca,Bryconameric
us_caucanus_76421_Magdalena_Cauca,Bryconamericus_caucanus_76423_Magdal
ena_Cauca,Bryconamericus_caucanus_76424_Magdalena_Cauca,Bryconamericus
_caucanus_76422_Magdalena_Cauca,Hemibrycon_boquiae_76529_Magdalena_cau
ca

Species 128 (support = 0.989)
Bryconamericus_iheringii_61391_Bsp_LaPlata_Uruguay

Species 129 (support = 0.989)
Bryconamericus_iheringii_34762_Bexo_LaPlata_Parana

Species 130 (support = 0.979)
'12-49367_Knodus_cf._delta_B'

Species 131 (support = 0.979)
'10-53834_Knodus_aff._megalops_C'

Species 132 (support = 0.990)
Bryconamericus_sp_34782_Bstr_Parana

Species 133 (support = 0.999)
Astyanax_daguae_76594_Dagua_SanCipriano

Species 134 (support = 0.999)
Bryconamericus_tenuis_79623_Parana_Tiete

Species 135 (support = 0.677)

Bryconamericus_alpha_76461_Orinoco,Bryconamericus_alpha_76459_Orinoco,
Knodus_sp5_76551_Orinoco_Meta

Species 136 (support = 0.726)

Knodus_spb_76569_Orinoco_Guaviare,Knodus_spb_76570_Orinoco_Guaviare,Knodus_spb_76572_Orinoco_Guaviare,Knodus_spb_76571_Orinoco_Guaviare,Knodus_sp1_76575_Orinoco_Guaviare,Knodus_spb_76573_Orinoco_Guaviare,Knodus_sp1_76574_Orinoco_Guaviare,Knodus_sp5_76552_Orinoco_Meta

Species 137 (support = 0.731)
Knodus_sp_76539_Orinoco_Meta,Knodus_sp_76540_Orinoco_Meta

Species 138 (support = 0.502)
Bryconamericus_gonzalezoi_76660_Changinola

Species 139 (support = 0.502)
Bryconamericus_gonzalezoi_76659_Changinola

Species 140 (support = 0.497)
Bryconamericus_exodon_22812_Parana_Paraguay

Species 141 (support = 0.497)
Bryconamericus_exodon_22811_Parana_Paraguay

Species 142 (support = 0.626)

Knodus_sp6_76566_Orinoco_Meta,Knodus_sp6_76567_Orinoco_Meta,Knodus_sp6_76568_Orinoco_Meta,Knodus_sp4_76555_Orinoco_Meta

Species 143 (support = 0.626)

Knodus_sp6_76557_Orinoco_Guaviare,Knodus_sp6_76542_Orinoco_Guaviare,Knodus_sp6_76543_Orinoco_Guaviare,Bryconamericus_macarenae_76479_Orinoco_Guaviare

Species 144 (support = 0.629)

Bryconamericus_cismontanus_76463_Orinoco,Bryconamericus_cismontanus_76467_Orinoco,Bryconamericus_cismontanus_76465_Orinoco,Bryconamericus_cismontanus_76466_Orinoco,Bryconamericus_cismontanus_76462_Orinoco,Bryconamericus_cismontanus_76464_Orinoco,Bryconamericus_cismontanus_76468_Orinoco

Species 145 (support = 0.666)
2-15818_Knodus_meridae

Species 146 (support = 0.517)
Bryconamericus_sp_76672_Amazonas

Species 147 (support = 0.517)
Bryconamericus_sp_76673_Amazonas

Species 148 (support = 0.754)

Bryconamericus_stramineus_22892_Parana,Bryconamericus_stramineus_22891_Parana,Bryconamericus_stramineus_Bsp_19675_Parana_Tiete

Species 149 (support = 0.631)

Bryconamericus_stramineus_34663_LaPlata_Paranapanema,Bryconamericus_stramineus_45605_Parana,Bryconamericus_stramineus_45606_Parana

Species 150 (support = 0.838)

Bryconamericus_tolimae_76427_Magdalena_Coello,Bryconamericus_tolimae_76440_Magdalena_Amoya,Bryconamericus_tolimae_76437_Magdalena_Amoya,Bryconamericus_tolimae_76430_Magdalena_Coello,Bryconamericus_tolimae_76428_Magdalena_Coello,Bryconamericus_tolimae_76429_Magdalena_Coello,Bryconamericus_tolimae_76426_Magdalena_Coello,Bryconamericus_tolimae_76439_Magdalena_Amoya,Bryconamericus_tolimae_76436_Magdalena_Amoya,Bryconamericus_tolimae_76438_Magdalena_Amoya,Bryconamericus_huilae_76434_Magdalena_Garzon,Bryconamericus_huilae_76435_Magdalena_Garzon,Bryconamericus_huilae_76432_Magdalena_Garzon,Bryconamericus_huilae_76431_Magdalena_Garzon,Bryconamericus_huilae_76433_Magdalena_Garzon

Species 151 (support = 0.946)

Bryconamericus_plutarcoi_76455_Magdalena_Fonce

Species 152 (support = 0.512)
Creagrutus_sp_76586_Amazonas_Caqueta

Species 153 (support = 0.512)
Creagrutus_sp_76587_Amazonas_Caqueta

Species 154 (support = 0.829)

Bryconamericus_spn_shibatta_Bsp_10995_LaPlata_Tibagi,Bryconamericus_spn_shibatta_Bexo_34763_Parana,Bryconamericus_spn_shibatta_Bexo_34659_LaPlata_Tibagi,Bryconamericus_spn_shibatta_Bsp_10996_LaPlata_Tibagi

Species 155 (support = 0.947)
Bryconamericus_ornaticeps_79627_Tingua

Species 156 (support = 0.711)

Bryconamericus_andresoi_76403_Patia_Timbio,Bryconamericus_andresoi_76402_Patia_Timbio,Bryconamericus_andresoi_76401_Patia_Timbio,Bryconamericus_andresoi_76405_Patia_Timbio,Bryconamericus_andresoi_76404_Patia_Timbio

Species 157 (support = 0.711)

Bryconamericus_galvisi_76412_Amazonas_Putumayo,Bryconamericus_galvisi_76413_Amazonas_Putumayo,Bryconamericus_galvisi_76411_Amazonas_Putumayo
