# Supplementary material for: Species delimitation of neotropical Characins (Stevardiinae): Implications for taxonomy of complex groups
Source: PLoS One. 2019 Jun 5;14(6):e0216786. doi: 10.1371/journal.pone.0216786 (PMC6550444; doi:10.1371/journal.pone.0216786)
Supplement: S4 File — The sequences allocated outside of Stevardiinae in the GMYC analysis, were removed. (DOCX) [file pone.0216786.s013.docx]

# Max likilhood partition

Species 1 (support = 1.000)

Bryconamericus_sp_34767_Bstr_Parana

Species 2 (support = 1.000)

Bryconamericus_tenuis_79622_Parana_Tiete

Species 3 (support = 1.000)

Bryconamericus_sp_38473_SaoFrancisco

Species 4 (support = 1.000)

4217243_Planaltina_britskii

Species 5 (support = 1.000)

Bryconamericus_sp_47611_Orinoco_Apure

Species 6 (support = 1.000)

Carlastyanax_aurocaudatus_76582_Magdalena_Cauca

Species 7 (support = 1.000)

133216_K_tiquiensis

Species 8 (support = 1.000)

527297_Knodus_sp_nov3

Species 9 (support = 1.000)

1817084_Bryconamericus_sp

Species 10 (support = 1.000)

4144042_Creagrutus_meridionalis20057perTanTs

Species 11 (support = 0.497)

Creagrutus_sp_76584_Magdalena_Pienta,Creagrutus_sp_76585_Magdalena_Pienta

Species 12 (support = 0.495)

Creagrutus_sp_76590_Orinoco_Guaviare,Creagrutus_sp_76589_Orinoco_Guaviare

Species 13 (support = 1.000)

4640243_Pseudocorynopoma_heterandia18570

Species 14 (support = 1.000)

44Corynopoma_riiseigenebank261825527

Species 15 (support = 1.000)

1454219_Knodus_aff_megalops_A

Species 16 (support = 1.000)

4938090_Lophiobrycon_weitzmani

Species 17 (support = 1.000)

5024541_Glandulocauda_melanopleuramelanogenis

Species 18 (support = 1.000)

657048_Knodus_spnov2

Species 19 (support = 0.935)

Bryconamericus_peruanus_43963_Tumbes,Bryconamericus_peruanus_45006_Tumbes,Bryconamericus_peruanus_43964_Tumbes,Bryconamericus_peruanus_43916_Zarumilla,Bryconamericus_peruanus_43915_Zarumilla

Species 20 (support = 0.411)

Bryconamericus_terrabensis_76675_Coto,Bryconamericus_terrabensis_76676_Coto,Bryconamericus_terrabensis_76679_Terraba,Bryconamericus_terrabensis_76678_Terraba,Bryconamericus_terrabensis_76677_Terraba

Species 21 (support = 1.000)

Bryconamericus_emperador_76606_Atrato

Species 22 (support = 1.000)

4821274_Mimagoniates_inequalis

Species 23 (support = 1.000)

4711077_Mimagoniates_microlepis

Species 24 (support = 1.000)

841554_Knodus_cf_chapadae_B

Species 25 (support = 0.505)

Creagrutus_sp_76586_Amazonas_Caqueta,Creagrutus_sp_76587_Amazonas_Caqueta

Species 26 (support = 0.801)

Knodus_sp2_76560_Orinoco_Guaviare,Knodus_sp2_76562_Orinoco_Guaviare,Knodus_sp1_76577_Orinoco_Guaviare,Knodus_sp2_76561_Orinoco_Guaviare

Species 27 (support = 0.559)

Bryconamericus_stramineus_34663_LaPlata_Paranapanema,Bryconamericus_stramineus_22891_Parana,Bryconamericus_stramineus_22892_Parana,Bryconamericus_stramineus_Bsp_19675_Parana_Tiete,Bryconamericus_stramineus_45606_Parana,Bryconamericus_stramineus_45605_Parana­

Species 28 (support = 0.736)

Bryconamericus_thomasi_76680_Parana,Bryconamericus_spn_canastra_Parg_47808_SaoFrancisco,Bryconamericus_pachacuti_79619_Amazonas_Ucayali,Bryconamericus_pachacuti_79620_Amazonas_Ucayali

Species 29 (support = 0.998)

913237_Knodus_breviceps_B

Species 30 (support = 0.999)

2744688_Knodus_sp2

Species 31 (support = 1.000)

Bryconadenos_tanaothoros_64727_Bsp_Amazonas_Xingu

Species 32 (support = 1.000)

2911815_Knodus_breviceps_A

Species 33 (support = 1.000)

2266362_K_cf_savannensis_A

Species 34 (support = 1.000)

1326478_Knodus_sp1

Species 35 (support = 1.000)

2657371_Knodus_heteresthes_A

Species 36 (support = 1.000)

1653759_Knodus_sp6

Species 37 (support = 1.000)

1753820_Knodus_borki

Species 38 (support = 1.000)

1923624_Knodus_cf_orteguasse_B

Species 39 (support = 1.000)

Bryconamericus_sp_16348_Amazonas

Species 40 (support = 0.999)

Knodus_sp_76538_Orinoco_Meta

Species 41 (support = 0.762)

Knodus_sp5_76552_Orinoco_Meta,Knodus_spb_76572_Orinoco_Guaviare,Knodus_spb_76571_Orinoco_Guaviare,Knodus_sp1_76575_Orinoco_Guaviare,Knodus_spb_76569_Orinoco_Guaviare,Knodus_sp1_76574_Orinoco_Guaviare,Knodus_spb_76570_Orinoco_Guaviare,Knodus_spb_76573_Orinoco_Guaviare,Bryconamericus_sp_76473_Orinoco_Guaviare,Bryconamericus_sp_76472_Orinoco_Guaviare,Knodus_sp_76540_Orinoco_Meta,Knodus_sp_76539_Orinoco_Meta

Species 42 (support = 0.660)

Bryconamericus_alpha_76596_Orinoco,Bryconamericus_alpha_76595_Orinoco,Knodus_sp5_76549_Orinoco_Meta

Species 43 (support = 0.506)

Bryconamericus_exodon_22811_Parana_Paraguay,Bryconamericus_exodon_22812_Parana_Paraguay

Species 44 (support = 0.995)

3838382_Piabarchus_analis

Species 45 (support = 0.697)

Bryconamericus_exodon_56523_Parana_Paraguay,Bryconamericus_exodon_56031_Parana_Paraguay,Bryconamericus_exodon_56032_Parana_Paraguay,Bryconamericus_exodon_55828_Parana_Paraguay,Bryconamericus_turiuba_35934_LaPlata_Paranaiba,Bryconamericus_turiuba_35933_LaPlata_Paranaiba,Bryconamericus_turiuba_36228_LaPlata_Paranaiba,Bryconamericus_turiuba_36227_LaPlata_Paranaiba,Bryconamericus_turiuba_55415_Bstr_Parana_Paraguay,Bryconamericus_turiuba_35935_LaPlata_Paranaiba,Bryconamericus_exodon_26192_Parana_Paraguay,Bryconamericus_exodon_55476_Parana_Paraguay,Bryconamericus_exodon_56378_Parana_Paraguay,Bryconamericus_exodon_56379_Parana_Paraguay,Bryconamericus_exodon_26191_Parana_Paraguay,Bryconamericus_exodon_56522_Parana_Paraguay

Species 46 (support = 0.496)

Bryconamericus_sp_76469_Orinoco_Guaviare,Bryconamericus_sp_76470_Orinoco_Guaviare

Species 47 (support = 0.672)

Bryconamericus_sp_46969_Orinoco,Bryconamericus_sp_76476_Orinoco_Meta,Bryconamericus_sp_46968_Orinoco

Species 48 (support = 0.820)

Bryconamericus_spn_shibatta_Bexo_34763_Parana,Bryconamericus_spn_shibatta_Bsp_10995_LaPlata_Tibagi,Bryconamericus_spn_shibatta_Bsp_10996_LaPlata_Tibagi,Bryconamericus_spn_shibatta_Bexo_34659_LaPlata_Tibagi,Bryconamericus_ornaticeps_79627_Tingua

Species 49 (support = 0.964)

Piabina_argentea_18788_Bsp_Parana_Tiete

Species 50 (support = 0.785)

Bryconamericus_ornaticeps_79628_Tingua,Bryconamericus_ornaticeps_79626_Tingua

Species 51 (support = 1.000)

3721306_Piabina_argentea

Species 52 (support = 0.051)

Bryconamericus_caucanus_76423_Magdalena_Cauca,Bryconamericus_caucanus_76424_Magdalena_Cauca,Bryconamericus_caucanus_76421_Magdalena_Cauca,Bryconamericus_caucanus_76422_Magdalena_Cauca,Hemibrycon_boquiae_76527_Magdalena_Cauca,Hemibrycon_boquiae_76526_Magdalena_Cauca,Hemibrycon_boquiae_76530_Magdalena_Cauca,Hemibrycon_boquiae_76528_Magdalena_Cauca,Hemibrycon_boquiae_76529_Magdalena_cauca,Bryconamericus_caldasi_76507_Magdalena_Cauca,Bryconamericus_caldasi_76506_Magdalena_Cauca,Bryconamericus_caldasi_76505_Magdalena_Cauca,Bryconamericus_caldasi_76504_Magdalena_Cauca,Bryconamericus_caldasi_76502_Magdalena_Cauca,Bryconamericus_caldasi_76503_Magdalena_Cauca,Bryconamericus_caucanus_76511_Magdalena_Cauca,Bryconamericus_caucanus_76509_Magdalena_Cauca,Bryconamericus_caucanus_76512_Magdalena_Cauca,Bryconamericus_caucanus_76510_Magdalena_cauca,Bryconamericus_andresoi_76404_Patia_Timbio,Bryconamericus_andresoi_76402_Patia_Timbio,Bryconamericus_andresoi_76403_Patia_Timbio,Bryconamericus_andresoi_76405_Patia_Timbio,Bryconamericus_andresoi_76401_Patia_Timbio,Bryconamericus_galvisi_76412_Amazonas_Putumayo,Bryconamericus_galvisi_76411_Amazonas_Putumayo,Bryconamericus_galvisi_76413_Amazonas_Putumayo

Species 53 (support = 0.963)

3333171_Odontostoechus_lethostigmus

Species 54 (support = 0.963)

Bryconamericus_iheringii_20482_Bsp_Atlantico

Species 55 (support = 0.567)

Bryconamericus_iheringii_61391_Bsp_LaPlata_Uruguay,Bryconamericus_iheringii_34199_LaPlata_Parana,Bryconamericus_iheringii_34198_Parana,3534200_Bryconamericus_exodon,Bryconamericus_iheringii_18706_Parana_Tiete,Bryconamericus_iheringii_18707_Parana_Tiete,Bryconamericus_iheringii_66564_Bika_Parana_Iguacu,Bryconamericus_iheringii_66563_Bika_Parana_Iguacu,Bryconamericus_iheringii_61324_Bsp_LaPlata_Uruguay,Bryconamericus_iheringii_68417_LaPlata_Uruguay,Bryconamericus_iheringii_34762_Bexo_LaPlata_Parana,Bryconamericus_iheringii_20483_Bsp_Atlantico,Bryconamericus_iheringii_21272_Atlantico,Bryconamericus_iheringii_21271_Atlantico,Bryconamericus_iheringii_21251_Bsp_Atlantico,Bryconamericus_iheringii_21252_Bsp_Atlantico

Species 56 (support = 0.998)

2337317_Knodus_heteresthes_B

Species 57 (support = 0.991)

1149366_Knodus_cf_delta_A

Species 58 (support = 0.970)

3625516_Cyanocharax_alburnus

Species 59 (support = 0.497)

Cyanocharax_itaimbe_60646_Bihe_Atlantico,Cyanocharax_itaimbe_60645_Bihe_Atlantico

Species 60 (support = 0.342)

Knodus_sp_74408_Bsp_Amazonas_Rionegro,Knodus_sp_74406_Bsp_Amazonas_RioNegro,Knodus_sp_74407_Bsp_Amazonas_RioNegro

Species 61 (support = 0.995)

723547_Knodus_cf_orteguasse_A

Species 62 (support = 0.901)

2066315_Knodus_sp5

Species 63 (support = 0.901)

2136223_Knodus_chapadae_C

Species 64 (support = 0.835)

Bryconamericus_emperador_76658_Dagua,Bryconamericus_emperador_76441_Dagua_Jesus,Bryconamericus_emperador_76442_Dagua_Jesus,Bryconamericus_emperador_76443_Dagua_Jesus

Species 65 (support = 0.617)

Bryconamericus_cismontanus_76465_Orinoco

Species 66 (support = 0.463)

Bryconamericus_cismontanus_76466_Orinoco

Species 67 (support = 0.846)

1053834_Knodus_aff_megalops_C

Species 68 (support = 0.846)

1249367_Knodus_cf_delta_B

Species 69 (support = 0.970)

1554223_Knodus_megalops_B

Species 70 (support = 0.478)

Knodus_hypopterus_76534_Amazonas_Caqueta,Knodus_hypopterus_76536_Amazonas_Caqueta

Species 71 (support = 0.967)

Bryconamericus_guaytarae_76419_Patia_Mojarras

Species 72 (support = 0.961)

4333168_Hemibrycon_taeniurus

Species 73 (support = 0.157)

Bryconamericus_cismontanus_76467_Orinoco,Bryconamericus_sp_47612_Orinoco_Apure,215818_Knodus_meridae­

Species 74 (support = 0.311)

Bryconamericus_cismontanus_76463_Orinoco

Species 75 (support = 0.328)

Bryconamericus_sp_76673_Amazonas,Bryconamericus_sp_76672_Amazonas

Species 76 (support = 0.678)

Bryconamericus_sp_34782_Bstr_Parana

Species 77 (support = 0.955)

Bryconamericus_scleroparius_76669_Changuinola

Species 78 (support = 0.336)

Bryconamericus_ichoensis_76489_Atrato_Icho,Bryconamericus_emperador_76487_Atrato_Icho,Bryconamericus_emperador_76484_Atrato_Icho,Bryconamericus_emperador_76486_Atrato_Icho,Bryconamericus_emperador_76483_Atrato_Icho,Bryconamericus_emperador_76485_Atrato_Icho

Species 79 (support = 0.377)

Bryconamericus_guaytarae_76408_Patia_Guachicono,Bryconamericus_guaytarae_76409_Patia_Guachicono

Species 80 (support = 0.341)

Knodus_moenkhausii_47707_Bexo_Parana,Knodus_moenkhausii_47689_Bexo_Parana,Knodus_moenkhausii_38496_Bsp_Jequitinhonha_Fanado,Knodus_moenkhausii_38498_Bsp_Jequitinhonha_Fanado,Knodus_moenkhausii_20342_Bsp_ParaibadoSul,Knodus_moenkhausii_20343_Bsp_ParaibadoSul,Knodus_moenkhausii_38497_Bsp_Jequitinhonha_Fanado,431936_Knodus_moenkhausii,Knodus_moenkhausii_47688_Bexo_Parana

Species 81 (support = 0.607)

Knodus_moenkhausii_17323_Bsp_Parana,Knodus_moenkhausii_47708_Bexo_Parana,Knodus_moenkhausii_17322_Bsp_Parana

Species 82 (support = 0.849)

Bryconamericus_ichoensis_76488_Atrato_Icho

Species 83 (support = 0.146)

Bryconamericus_emperador_76651_SanJuan,Bryconamericus_emperador_76652_SanJuan,Bryconamericus_emperador_76653_SanJuan,Bryconamericus_bayano_76597_Sindatos,Bryconamericus_emperador_76664_SanJuan,Bryconamericus_emperador_76633_Tuira_Yape,Bryconamericus_emperador_76634_Tuira_Yape,Bryconamericus_emperador_76621_PlayonChico,Bryconamericus_emperador_76631_Azucar,Bryconamericus_emperador_76630_Azucar,Bryconamericus_emperador_76632_Azucar,Bryconamericus_emperador_76608_Mandinga,Bryconamericus_emperador_76607_Mandinga,Bryconamericus_emperador_76648_Anton,Bryconamericus_emperador_76642_Calovevora,Bryconamericus_emperador_76643_Calovevora,Bryconamericus_emperador_76644_Calovevora,Bryconamericus_emperador_76625_Bayano,Bryconamericus_emperador_76626_Bayano,Bryconamericus_emperador_76610_CocledelNorte,Bryconamericus_emperador_76611_CocledelNorte,Bryconamericus_emperador_76612_CocledelNorte,Bryconamericus_emperador_76618_Pato,Bryconamericus_emperador_76619_Pato,Bryconamericus_emperador_76637_Cascajal,Bryconamericus_emperador_76656_PinaPina,Bryconamericus_emperador_76654_PinaPina,Bryconamericus_emperador_76657_PinaPina,Bryconamericus_emperador_76655_PinaPina,Bryconamericus_emperador_18525_Atlantico,Bryconamericus_emperador_76638_Caimito,Bryconamericus_emperador_76640_Caimito,Bryconamericus_emperador_18524_Atlantico,Bryconamericus_emperador_76639_Caimito,Bryconamericus_emperador_76645_Chagres,Bryconamericus_emperador_76646_Chagres,Bryconamericus_emperador_76647_Chagres,Bryconamericus_emperador_76614_MigueldelaBorda,Bryconamericus_emperador_76613_MigueldelaBorda,Bryconamericus_emperador_76627_Indio,Bryconamericus_emperador_76628_Indio,Bryconamericus_emperador_76629_Indio

Species 84 (support = 0.933)

Knodus_sp6_76559_Orinoco_Guaviare

Species 85 (support = 0.498)

Bryconamericus_alpha_76459_Orinoco,Bryconamericus_alpha_76461_Orinoco,Knodus_sp5_76551_Orinoco_Meta,Knodus_sp6_76557_Orinoco_Guaviare,Knodus_sp6_76542_Orinoco_Guaviare,Bryconamericus_macarenae_76479_Orinoco_Guaviare,Knodus_sp6_76543_Orinoco_Guaviare,Knodus_sp4_76555_Orinoco_Meta,Knodus_sp6_76568_Orinoco_Meta,Knodus_sp6_76566_Orinoco_Meta,Knodus_sp6_76567_Orinoco_Meta

Species 86 (support = 0.788)

Bryconamericus_guaytarae_76407_Patia_Guachicono

Species 87 (support = 0.246)

Bryconamericus_plutarcoi_76455_Magdalena_Fonce,Bryconamericus_huilae_76433_Magdalena_Garzon,Bryconamericus_huilae_76431_Magdalena_Garzon,Bryconamericus_huilae_76434_Magdalena_Garzon,Bryconamericus_huilae_76432_Magdalena_Garzon,Bryconamericus_huilae_76435_Magdalena_Garzon,Bryconamericus_tolimae_76427_Magdalena_Coello,Bryconamericus_tolimae_76437_Magdalena_Amoya,Bryconamericus_tolimae_76428_Magdalena_Coello,Bryconamericus_tolimae_76440_Magdalena_Amoya,Bryconamericus_tolimae_76426_Magdalena_Coello,Bryconamericus_tolimae_76429_Magdalena_Coello,Bryconamericus_tolimae_76430_Magdalena_Coello,Bryconamericus_tolimae_76436_Magdalena_Amoya,Bryconamericus_tolimae_76438_Magdalena_Amoya,Bryconamericus_tolimae_76439_Magdalena_Amoya

Species 88 (support = 0.389)

Bryconamericus_macarenae_76478_Orinoco_Guaviare,Bryconamericus_macarenae_76480_Orinoco_Guaviare

Species 89 (support = 0.985)

2427342_Knodus_victoriae

Species 90 (support = 0.985)

2544636_Knodus_sp4

Species 91 (support = 0.297)

Bryconamericus_arilepis_76446_Magdalena_Fonce,Bryconamericus_arilepis_76447_Magdalena_Fonce,Bryconamericus_arilepis_76448_Magdalena_Fonce,Bryconamericus_arilepis_76444_Magdalena_Fonce,Bryconamericus_arilepis_76445_Magdalena_Fonce,Bryconamericus_plutarcoi_76453_Magdalena_Fonce,Bryconamericus_plutarcoi_76454_Magdalena_Fonce,Bryconamericus_plutarcoi_76456_Magdalena_Fonce

Species 92 (support = 0.318)

Bryconamericus_guaytarae_76417_Patia_Mojarras,Bryconamericus_guaytarae_76420_Patia_Mojarras

Species 93 (support = 0.745)

3062500_Knodus_cf_savanensis_B

Species 94 (support = 0.442)

Knodus_sp_43802_Bsp_Amazonas_Guama,Knodus_sp_43803_Bsp_Amazonas_Guama,3243070_Knodus_spnov1,3116318_Knodus_sp3,Knodus_sp_13340_Bsp_Amazonas_Araguaia,Knodus_sp_22843_Bsp_Tocantins_Araguaia,Knodus_sp_22844_Bsp_Tocantins_Araguaia,Knodus_sp_13339_Bsp_Amazonas_Araguaia,2827521_Knodus_cf_chapadae_A

Species 95 (support = 0.791)

Bryconamericus_tenuis_79623_Parana_Tiete

Species 96 (support = 0.791)

Astyanax_daguae_76594_Dagua_SanCipriano

Species 97 (support = 0.506)

Bryconamericus_gonzalezoi_76659_Changinola

Species 98 (support = 0.506)

Bryconamericus_gonzalezoi_76660_Changinola

Species 99 (support = 0.323)

Bryconamericus_guaytarae_76406_Patia_Guachicono

Species 100 (support = 0.323)

Bryconamericus_guaytarae_76416_Patia_Mojarras

Species 101 (support = 0.606)

Bryconamericus_sp_76496_Dagua_SanCipriano

Species 102 (support = 0.703)

Bryconamericus_foncensis_76451_Magdalena_Pienta

Species 103 (support = 0.396)

Bryconamericus_macarenae_76481_Orinoco_Guaviare

Species 104 (support = 0.396)

Bryconamericus_macarenae_76482_Orinoco_Guaviare

­­Species 105 (support = 0.663)

3433174_Hypobrycon_maromba

Species 106 (support = 0.513)

Bryconamericus_sp_76500_Dagua_SanCipriano

Species 107 (support = 0.092)

Bryconamericus_sp_76499_Dagua_SanCipriano,Bryconamericus_sp_76497_Dagua_SanCipriano,Bryconamericus_sp_76495_Dagua_SanCipriano,Bryconamericus_sp_76501_Dagua_SanCipriano,Bryconamericus_sp_76498_Dagua_SanCipriano

Species 108 (support = 0.481)

Bryconamericus_foncensis_76452_Magdalena_Pienta

Species 109 (support = 0.241)

Bryconamericus_foncensis_76450_Magdalena_Pienta

Species 110 (support = 0.241)

Bryconamericus_foncensis_76449_Magdalena_Pienta

Species 111 (support = 0.501)

Bryconamericus_brevirrostris_76599_Tumbes

Species 112 (support = 0.501)

Bryconamericus_brevirrostris_76598_Tumbes

Species 113 (support = 0.499)

Bryconamericus_peruanus_76665_Canete

Species 114 (support = 0.499)

Bryconamericus_peruanus_76666_Canete

Species 115 (support = 0.794)

Hemibrycon_metae_76523_Orinoco_Guaviare

Species 116 (support = 0.164)

Hemibrycon_metae_76522_Orinoco_Guaviare,Hemibrycon_metae_76521_Orinoco_Guaviare,Hemibrycon_metae_76524_Orinoco_Guaviare,Knodus_sp1_76578_Orinoco_Guaviare,Hemibrycon_metae_76525_Orinoco_Guaviare

Species 117 (support = 0.333)

Bryconamericus_iheringii_54928_Bpat_LaPlata_Uruguay

Species 118 (support = 0.333)

Bryconamericus_iheringii_54927_Bpat_LaPlata_Uruguay

Species 119 (support = 0.379)

Bryconamericus_guaytarae_76418_Patia_Mojarras

Species 120 (support = 0.379)

Bryconamericus_guaytarae_76410_Patia_Guachicono

Species 121 (support = 0.749)

Hemibrycon_divisorensis_76513_Amazonas_Caqueta

Species 122 (support = 0.523)

Bryconamericus_ornaticeps_79629_Tingua

Species 123 (support = 0.523)

Bryconamericus_ornaticeps_79630_Tingua

Species 124 (support = 0.664)

363123_Bryconamericus_diaphanus

Species 125 (support = 0.501)

Hemibrycon_divisorensis_76517_Amazonas_Caqueta

Species 126 (support = 0.250)

Hemibrycon_divisorensis_76515_Amazonas_Caqueta,Hemibrycon_divisorensis_76516_Amazonas_Caqueta

Species 127 (support = 0.326)

Bryconamericus_diaphanus_63123_Amazonas

Species 128 (support = 0.326)

Bryconamericus_diaphanus_63124_Amazonas

Species 129 (support = 0.249)

Bryconamericus_cismontanus_76468_Orinoco,Bryconamericus_cismontanus_76462_Orinoco

Species 130 (support = 0.516)

Bryconamericus_cismontanus_76464_Orinoco

Species 131 (support = 0.281)

Hemibrycon_raqueliae_76533_Magdalena,Hemibrycon_raqueliae_76531_Magdalena,Hemibrycon_raqueliae_76532_Magdalena

Species 132 (support = 0.816)

Bryconamericus_multiradiatus_76475_Atrato_Leon
